# Climate and land-use as the main drivers of recent environmental change in a mid-altitude mountain lake, Romanian Carpathians

**DOI:** 10.1371/journal.pone.0239209

**Published:** 2020-10-01

**Authors:** Aritina Haliuc, Krisztina Buczkó, Simon M. Hutchinson, Éva Ács, Enikő K. Magyari, Janos Korponai, Robert-Csaba Begy, Daniela Vasilache, Michal Zak, Daniel Veres

**Affiliations:** 1 Department of Atmospheric Physics, Faculty of Mathematics and Physics, Charles University, Praga, Czech Republic; 2 Romanian Young Academy, Research Institute of the University of Bucharest, University of Bucharest, Bucharest, Romania; 3 Centre for Ecological Research, GINOP Sustainable Ecosystems Group, Tihany, Hungary; 4 EPOC, UMR 5805, Université de Bordeaux, Pessac, France; 5 Department of Botany, Hungarian Natural History Museum, Budapest, Hungary; 6 MTA Centre for Ecological Research, Danube Research Institute, Budapest, Hungary; 7 School of Science, Engineering and Environment, University of Salford, Salford, United Kingdom; 8 Faculty of Water Sciences, National University of Public Service, Baja, Hungary; 9 MTA-MTM-ELTE Research Group for Paleontology, Department of Environmental and Landscape Geography, Eötvös Lorand University, Budapest, Hungary; 10 Department of Environmental Sciences, Sapientia Hungarian University of Transylvania, Cluj-Napoca, Romania; 11 Interdisciplinary Research Institute on Bio-Nano-Science, Babes-Bolyai University, Cluj-Napoca, Romania; 12 Faculty of Environmental Science and Engineering, Babeş-Bolyai University, Cluj-Napoca, Romania; 13 Romanian Academy, Institute of Speleology, Cluj-Napoca, Romania; Institute of Tibetan Plateau Research Chinese Academy of Sciences, CHINA

## Abstract

Recent decades have been marked by unprecendented environmental changes which threaten the integrity of freshwater systems and their ecological value. Although most of these changes can be attributed to human activities, disentagling natural and anthropogenic drivers remains a challenge. In this study, surface sediments from Lake Ighiel, a mid-altitude site in the Carpathian Mts (Romania) were investigated following high-resolution sedimentological, geochemical, environmental magnetic and diatom analyses supported by historical cartographic and documentary evidence. Our results suggest that between 1920 and 1960 the study area experienced no significant anthropogenic impact. An excellent correspondence is observed between lake proxy responses (e.g., growth of submerged macrophytes, high detrital input, shifts in diatom assemblages) and parameters tracking natural hydroclimate variability (e.g., temperature, NAO). This highlights a dominant natural hydroclimatic control on the lacustrine system. From 1960 however, the depositional regime shifted markedly from laminated to homogenous clays; since then geochemical and magnetic data document a trend of significant (and on-going) subsurface erosion across the catchment. This is paralleled by a shift in lake ecosystem conditions denoting a strong response to an intensified anthropogenic impact, mainly through forestry. An increase in detrital input and marked changes in the diatom community are observed over the last three decades, alongside accelerated sedimentation rates following enhanced grazing and deforestation in the catchment. Recent shifts in diatom assemblages may also reflect forcing from atmospheric nitrogen (N) deposition, a key recent drive of diatom community turnover in mountain lakes. In general, enhanced human pressure alongside intermittent hydroclimate forcing drastically altered the landscape around Lake Ighiel and thus, the sedimentation regime and the ecosystem’s health. However, paleoenvironmental signals tracking natural hydroclimate variability are also clearly discernible in the proxy data. Our work illustrates the complex link between the drivers of catchment-scale impacts on one hand, and lake proxy responses on the other, highlighting the importance of an integrated historical and palaeolimnological approach to better assess lake system changes.

## Introduction

Anthropogenic activities such as changes in land-use can induce major transformations in lake systems via increased catchment erosion, and its effect on sedimentation rates and nutrient loads leading to eutrophication and ecological shifts affecting lake biota [1–5]. Tracing such environmental dynamics over short timescales and assessing the type and timing of the main drivers of change are needed for a better understanding of the complex cause-effect relationship between environmental responses, anthropogenic activities and natural climate variability, and therefore to improve management strategies [6–8].

A large body of research suggests that recent anthropogenic activities greatly altered lake systems health via enhanced sediment input from watershed erosion and significant biogeochemical disturbances related to the widespread use of fertilisers and fossil fuel combustion [9–12]. A prevailing view is that, although millennia old anthropogenic activities are traceable in paleolimnological proxies, these earlier changes were rather local and of low intensity and consequently did not necessarily cause major shifts in aquatic ecosystems [13 and references there in]. However, a recent landscape-scale paleolimnological synthesis [11], highlighted the relative roles played by various driving factors behind the current rate of change affecting lake ecosystems globally. This study generally indicated that human-driven soil erosion was already ubiquitous 4,000 years ago following deforestation that induced enhanced rates of sediment transfer at a global scale [11].

The first detectable signs of significant anthropogenic impact over south-eastern Europe are traceable back to the Late Neolithic circa 7500 years ago, following the early advent of agriculture [14], shifts in land-use [15] and metal processing in this region [16]. Furthermore, as the Carpathian area sits at the junction of three major atmospheric pressure systems in Europe, the Atlantic, Mediterranean and Siberian High [17], retrieving reliable paleolimnological data from natural archives in this region underscores the need to reliably disentangle natural hydroclimate forcing on one hand [18–21] from longer term anthropogenic signals on the other [22, 23]. It has been shown that even for relatively remote high-altitude environments in the Carpathians, recent human activities have greatly altered landscape stability and thereby the lacustrine depositional regimes [24, 25]. However, more data are needed for a compelling view on the current rate of change. Therefore, most recent short-time frame paleoenvironmental data (pollen, sediment accumulation rates, geochemistry) may not necessarily provide a reliable background reference for interpreting long-term natural climate variability in the Carpathians [15, 22, 26, 27], as similarly documented for the Alps [28] and elsewhere [13]. However, such data can provide significant information on the current state of environment conservation and might provide hints on the future trajectories of change.

In this study we focus on Lake Ighiel, a mid-altitude site from the Apuseni Mountains (Romania) and one of the few natural records from the Romanian Carpathians providing high-resolution (paleo)environmental and (paleo)climatic data for the last 6000 years [18]. Previous work [18] showed that on a long-term perspective, the recent environmental changes experienced by the Ighiel catchment are outstanding and unprecedented in magnitude. Using two short cores covering the past eight decades we aim to explore, at the highest resolution possible, these recent environmental changes, to disclose the main drivers (documented hydroclimatic and anthropogenic impacts) and evaluate their short-term impacts on the catchment and lake ecosystem. Based on this dataset we evaluate Lake Ighiel’s main environmental stressors and advice on the best restoration targets and management guidelines for protecting this valuable ecological hot spot [29, 30].

## Regional setting

**Fig 1:**
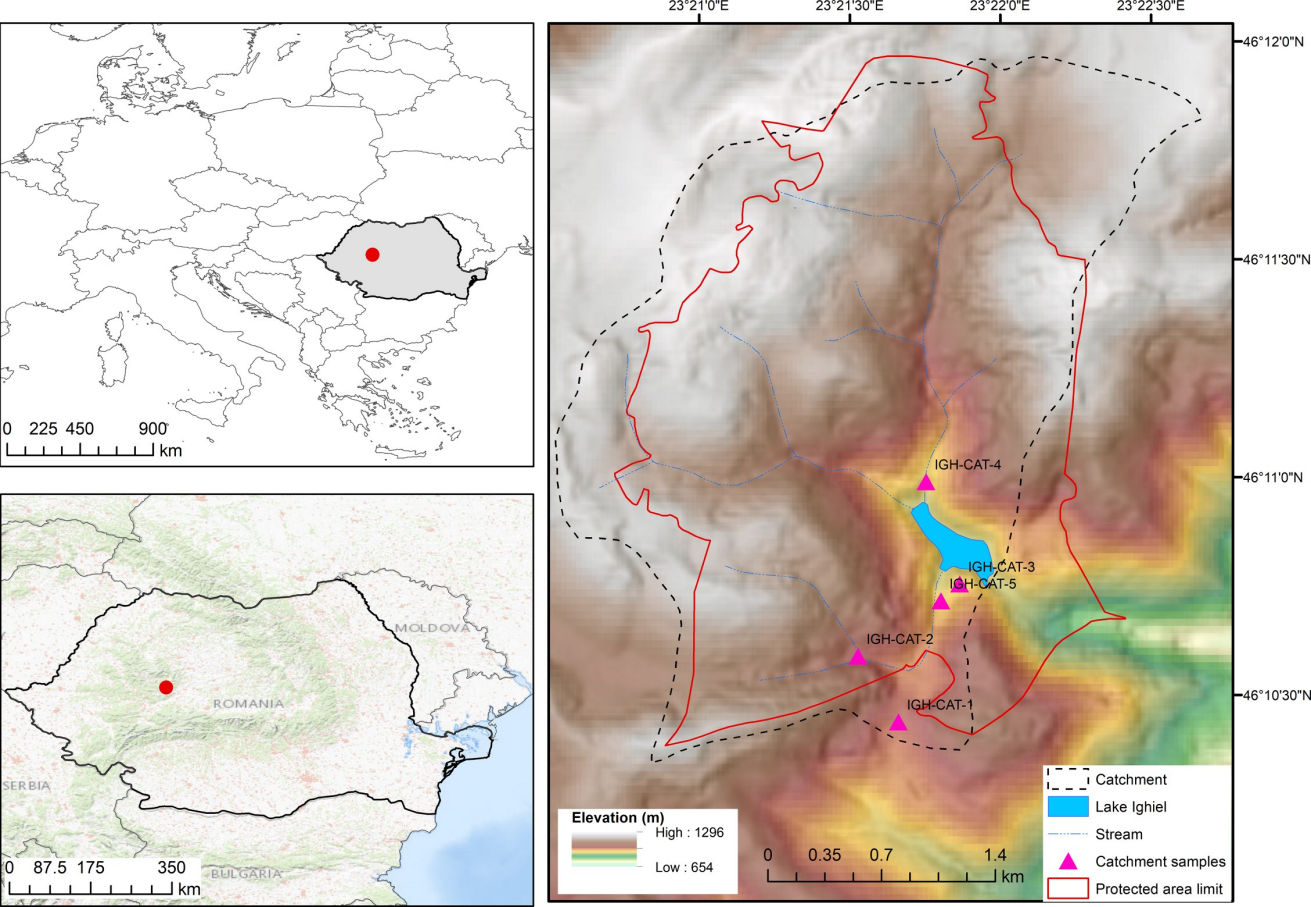
**The location of study area within Europe and Romania (left side).** A digital elevation model of Lake Ighiel catchment with temporary tributaries (tributaries as shown on topographic map 1:25000, 1978), the perimeter of protected area and soil sample locations (Reprinted from ALOS digital surface model (AW3D30) under a CC BY license, with permission from JAXA, original copyright [2020]).

Lake Ighiel (924 m a.s.l.; 46°10'50"N, 23°22'00"E) has a catchment area of 381 ha, a 3.20 ha water surface and a 8–9 m water depth [18]. The lake is highly sensitive to seasonal changes in water supply registering a maximum in spring and a minimum in autumn-winter with lake level changes of up to 3 m (S4 Fig). Since 1969 Lake Ighiel has been listed as natural reserve and a protected area of national interest (IV Category, IUCN) since 2000. The lake and catchment should be under a protected regime restricting detrimental environmental activities in the surrounding 365 ha buffer zone; however, these legal prerogatives are not enforced (Fig 1).

Two intermittent tributaries Striglau and Plesanului (Fig 1) discharge into the lake during high rainfall. In the 1980 hydro-technical works were undertaken to stabilise the riverine network. It appears, however, that these works, and the continuation of forestry, have resulted in even more drastic alteration of natural conditions in the buffer zone. Recent assessments of living biota and the ecological status of the water column [29, 30] indicate the risk of eutrophication unless measures are taken to limit anthropogenic organic pollution.

The local bedrock comprises mainly Mesozoic limestones and a band of diabaze rocks outcrop near the lake. The soil cover comprises cambisols (eu-mesobasic brown soils) and mollisols (rendzina). At present the catchment is mostly covered by deciduous forest with beech (*Fagus sylvatica*) and hornbeam (*Carpinus betulus*) as the dominant species, whereas the deforested areas within the upper catchment are kept open as pastures (Fig 1).

Mean annual temperatures range between 5–7.5°C at the Cluj-Napoca meteorological station (70 km to the north). In the Lake Ighiel area rainfall reaches 800–1000 mm/year and is concentrated mainly between May and August.

## Material and methods

### Sediment coring and soil profiles

Two short cores SC-3 (96 cm long) and SC-4 (88 cm long) were collected in autumn 2014 using a modified gravity corer with permission of Romsilva and Administratia Siturilor Natura 2000 Trascău. The cores were cut in half longitudinally, described, photographed and stored at ~4ºC. In order to assess the main sediment delivery pathways, five soil profiles (40 to 100 cm in depth) and 11 surface soil and bedrock samples were collected from the catchment area reflecting different geological substratum, land-cover and distance from the lake (Fig 1).

### Geochemical and mineral magnetic analyses

High-resolution geochemical screening was performed using an Itrax X-ray fluorescence core-scanner Cox Ltd applied to freshly split cores at the GFZ Laboratory, Potsdam. The running settings comprised a 1 mm resolution, 15 s exposure time, 40 kV tube voltage and a 40 mA tube current [31]. The geochemical data have been normalized using the coherent/incoherent (COH/INC) ratio to reduce impact from matrix effects [32].

Volume magnetic susceptibility (κ) readings were undertaken at 1 cm resolution on both the sediment cores and soil profiles and analyzed using a Bartington Instruments Ltd MS2B sensor. Organic matter (OM), inorganic carbon (IC) and minerogenic matter (MM) were determined on contiguously 1 cm sediment samples using loss-on-ignition [33] and expressed as percentage (%) of the sediment dry weight.

Particle size analyses were performed on ashed and carbonate-free sediment samples using a Horiba Laser Scattering Particle Size Analyzer (Partica LA-950). To reduce uncertainties, each sample was measured in three times following 1 minute ultrasonication. The samples were collected at 3 cm resolution. Here we use the median particle size distribution (D 50) as an indicator of erosional activity in the catchment [34].

### Chronology

**Fig 2:**
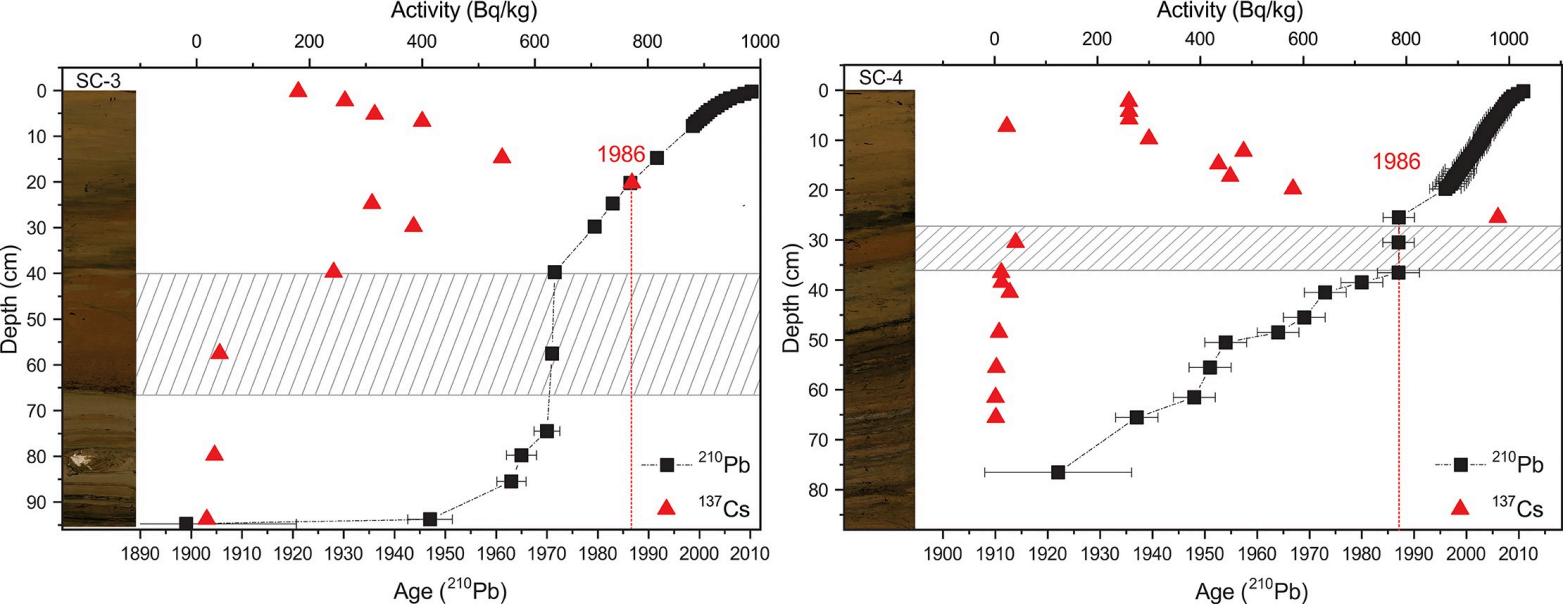
Age-depth model based on ^210^Pb and ^137^Cs for SC-3 and SC-4 sediment cores alongside core photos. ^*137*^Cs and ^*210*^Pb concentrations are plotted. Turbidites are marked with diagonal hatching.

The age model was constructed based on radiogenic decay dating for both SC-3 (27 samples) and SC-4 (53 samples) (Fig 2, S1 Table). Concentrations of ^210^Pb, ^226^Ra and ^137^Cs were determined employing a Be window HPGe detector (Ortec GMX). Standard materials of the same matrix and geometry (IAEA-327, 312, 375) were used for source calibration. ^226^Ra was measured after a month of sample storage (to reach the equilibrium between ^226^Ra/^222^Rn and its short half-life radionuclides ^214^Pb and ^214^Bi). For the determinations: 46.5 keV for ^210^Pb, 295 and 351 keV for ^214^Pb and for ^137^Cs the 661 keV gamma lines were used. The relative 2σ uncertainty of measurements was below 20%; due to low activities of ^210^Pb in deeper sediment layers, ^210^Po was targeted instead, and measured by alpha spectrometry. For this purpose, an aliquot of 0.5g of sediment was digested in mineral acids (HNO_3_, HCl, H_2_O_2_) followed by spontaneous deposition of the ^210^Po radionuclide on a stainless-steel disc (with high Ni content).

To account for non-constant sedimentation processes, for both SC-3 and SC-4 records the Constant Rate of Supply (CRS) model was used for deriving a reliable age-depth model [35]. The identified turbidites (see discussion in Chapter 4) were excluded from the age model (Fig 2, S1 Table).

### Diatom analyses

In total, 84 samples from core SC-4 were analyzed for siliceous algae after preparation by standard cleaning methods [36]. Taxonomy mainly follows [37] and at least 400 valves were counted per sample using light microscopy (Leica DM LB2 equipped with 100 HCX PLAN APO object-lens) except the samples between 25–36 cm and 38–41 cm depth where less than 400 valves were identified. The taxonomical position of the diatoms was confirmed with Algaebase [38]. The constrained incremental sum of squares (CONISS) method was used to highlight major changes in the diatom record and separate diatom stratigraphic zones, on square-root transformed data. Based on habitat preference we classified the siliceous algae into four groups: (1) aerophytic, (2) benthic, (3) periphytic, and (4) planktic taxa [39]. The turbidite layer at 25–36 cm was excluded from further evaluation, whereas diatom data for the interval 38–41 cm depth are included. However, this part of the record must be interpreted with caution as the diatom valve number was low.

### Historical maps and documents

Three sets of historical topographic maps were used to evaluate changes in regional forest cover at reference points in time. The oldest available is the second Franciscan military survey of the Habsburg Empire compiled between 1853–1858 and 1869–1870 at 1:28800 resolution [40]. Although its clarity is rather low, the details and color codes helped securely identify at least areas with no forest vegetation at the time of the survey. As a simplification, we used 1870 as last year of survey for referencing the map. The other cartographic resources include the 1:20000 military plans from 1957 and the 1:25000 Romanian military topographic field survey map (second edition) of 1974–1978 (for simplification referenced here to the last year of survey, 1978). The non-forest areas including pasture, pasture with scattered woody vegetation, and surfaces without vegetation were manually digitized in ArcGIS Pro 2.3.

**Fig 3:**
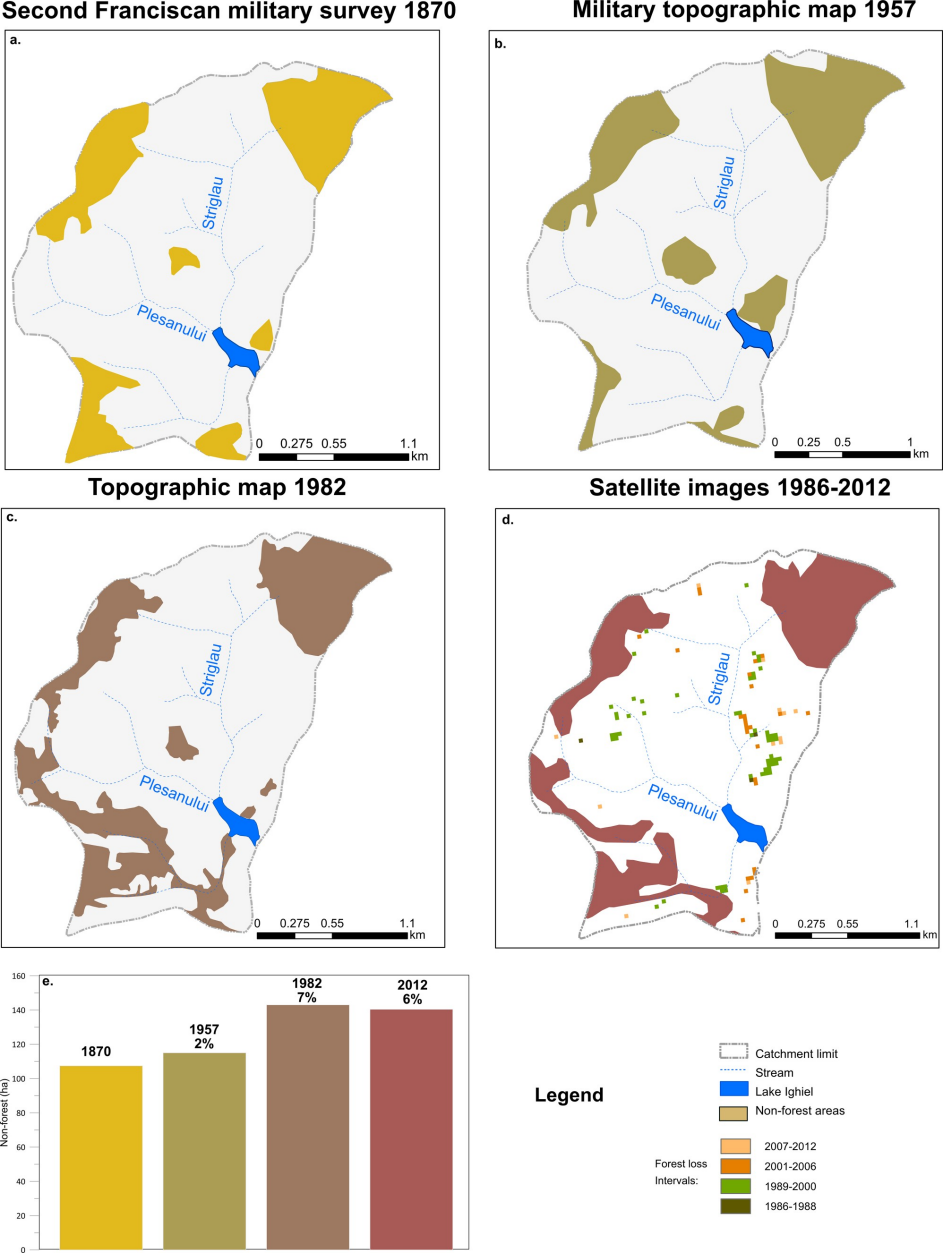
Changes in non-forest area at reference points in time using cartographic resources from a) 1870, b) 1957, c) 1978 and d) Landsat imagery from 1986–2012 [41] (European Environment Agency 2012). e) Non-forest area (ha) for each point in time relative to the total non-forest over the study period (1870–2012) and forest loss percentage (%) relative to 1870.

In addition to historical maps, we used the Landsat-based forest loss estimates from [41] covering the interval 1986–2012 to highlight the forest loss at catchment level over four contiguous intervals: 1986–1988, 1989–2000, 2001–2006 and 2007–2012, respectively (Fig 3). For the Landsat dataset we adopted the forest loss date codes (intervals) from [41]. Furthermore, we used the most recent (2012) Corine Land Cover (CLC) dataset [42] to document changes in land-use, which clearly registered the extensive forest loss, especially on the NE and SW parts of the catchment. However, given the different units used, these later estimations cannot be directly compared with those from the historical maps, but they can help form a more complete picture of recent land-use changes.

Information regarding the population size of Transylvania was extracted from census data [43] while number of inhabitants for Alba county was extracted from the national data census [44]. This estimation offers a regional image of recent changes in local and regional inhabitant numbers.

### Hydroclimate data

The mean annual rainfall for the Cluj-Napoca meteorological station was retrieved from the European Climate Assessment Project as the closest long-term meteorological station to the site [45]. Temperature anomaly data were downloaded from [46]. The self-calibrating Palmer Drought Severity Index (scPDSI), a parameter for dryness [47, 48] and North Altantic Oscillation Index (NAO) data were retrived from the CRU dataset [49] using the grid which intersects our study area. The Atlantic Mutidecadal Oscillation/Variability (AMO/AMV) data was retrieved from NOAA ESRL Physical Sciences Division (PSD) [50].

### Data treatment

Redundancy analysis (RDA) was used to explore the relationship between changes in lake biota as expressed by variability in the diatom assemblages and the other environmental proxies. This analysis was chosen because the gradient lengths of the first axes were less than four standard deviation (SD) units [51]. Statistical analyses were performed using R-software [52] with Vegan [53] and Rioja [54] packages.

The response variables include 136 diatom taxa, while sediment physical and geochemical proxies (Ti, K, Fe, Ca, OM—organic matter, magnetic susceptibility) and hydroclimate-related parameters (ScPDSI, precipitation, AMO, NAO, temperature anomaly) were employed as potential explanatory environmental variables. In the environmental matrix, data were averaged every cm using a 0.5 mid-point and transformed using square-root transformation and standardisation. We performed this analysis excluding the turbidites. The significant explanatory variables were selected by stepwise selection using the ordistep procedure of Vegan package. Variables which were not significant, or those showing high multicollinearity on variance infliction factor test (VIF> 20) and rare diatom taxa (percentage <2%), were removed from the analysis. Notwithstanding, the application of RDA to investigate the geochemical/physical and hydroclimate parameters was not possible given the very high correlation between the geochemical proxies.

## Results and interpretation

### Age-depth model

The age-depth models were calculated using the CRS (constant rate of ^210^Pb supply) dating model [35, 55]. In core SC-4, 1986, Chernobyl radiation peak, was placed at 25.5 cm depth in agreement with the ^137^Cs data (Fig 2). For SC-3, 1986 is recorded at 20 cm depth, which overlaps with the depth suggested by ^137^Cs activity. Turbidites were identified between 36–25 cm in SC-4 and 70–40 cm in SC-3 (S1 Table). These events exhibit different sedimentological characteristics between the two cores and reflect different events in time; this inference is also supported by the dating results (Fig 2) and diatom analysis (unpublished data for SC-3). As the turbidite is thinner in SC-4, we used this core as a key record for our high-resolution analyses. In this core, calculated sedimentation rates indicate a peak in accumulation between 1937 and 1948, followed by a smaller peak in 1964. Between 1995 and 2007 a highly variable trend is seen, but with a consistent increase and sediment accumulation rate (SAR) values between 0.3–1 g/cm^2^/yr^-1^ (Fig 2). SAR is used here as a physical parameter, which expresses changes in sediment input as a result of accelerated soil catchment erosion [56].

### Soil profiles and catchment sediment samples

Five soil profiles and eleven individual soil/rock samples were collected from the catchment area (S1 Text and S3 Fig) and logged for volume magnetic susceptibility (κ) to fingerprint the potential magnetic signature of the main sediment source areas and estimate their potential contribution to in-lake sedimentation by comparison with κ data from cores SC-3 and SC-4 (Table 1).

**Table 1:** Soil profiles, soil and bedrock samples collected at selected locations (see Fig 1) from Lake Ighiel’s catchment area.

Fieldwork ID	Type	Coordinates		Location	Length	Depth	κ (10^−5^ SI)
		Latitude	Longitude				
**IGH-CAT-1**	**Profile**	46.174305	23.360744	Upper pastureland south	100 cm	<20 cm	11
						>20 cm	22
IGH-CAT-1-Forest road clay	Sample						4.15
**IGH-CAT-2.1**	**Profile**	46.176186	23.358368	Upper south part close to forest	60 cm	<15 cm	29
						>15 cm	56
**IGH-CAT-2.2**	**Profile**	46.1761924	23.3585560	Upper south, ravine	95 cm	>15 cm	30
						>15 cm	86
**IGH-CAT-2.3**	**Profile**	46.176217	23.358485	Upper south, ravine	98 cm	Top 20 cm disturbed	-
						>20 cm	110
IGH-CAT-2- Forest road red clay	Sample						53.56
**IGH-CAT-3**	**Profile**	46.179511	23.364189	Lower, forested slope close to lake	36 cm	<20 cm	27
						>20 cm	51
**IGH-CAT-4**	**Profile**	46.183239	23.362459	Lower Striglau channel, forested	82 cm	<20 cm	22
						>20 cm	32
IGH-CAT-4-Small clay pellets	Sample						6.26
IGH-CAT-4-Big clay pellets	Sample						0.50
IGH-CAT-4-Limestone	Sample						-0.11
IGH-CAT-4-Sandstone	Sample						0.58
IGH-CAT-4-Fine-grained slope-wash, well sorted material	Sample						27.42
IGH-CAT-4-Coarse-grained slope-wash material	Sample						61.67
**IGH-CAT-5**	**Profile**	46.178840	23.363648	Lower pastureland south close to lake	100 cm	<20 cm	11
						>20 cm	15
IGH-CAT-5-Fine grained slope-wash, well-sorted	Sample						10.64
IGH-CAT-5-Coarse grain slope-wash material	Sample						13.19
IGH-CAT-5-Sandstone	Sample						5.92

The fieldwork ID, type of profile (soil profiles are in bold), coordinates, location description, profile depth, sampled depth interval and volume magnetic susceptibility are presented.

The main soil types include gleysols found mainly under the southern pasturelands (soil profiles IGH-CAT-1, IGH-CAT-5), cambisols along the northern Striglau channel banks (IGH-CAT-2.1 to 2.3, IGH-CAT-4), while umbrisols outcrop (IGH-CAT-3) in the proximity of the lake in the forested area (Fig 1).

Overall, the κ behaviour of the soil profiles shows that lower κ values characterize erosion of the top-soil Ah horizon, while high κ values reflect basal erosion as currently documented in over deepened catchment channels and ravines draining towards the lake (for more details please see S4 and S5 Figs). The relatively low κ values for the IGH-CAT-1 and IGH-CAT-5 soil profiles likely reflect gleization, a chemical alteration of magnetic minerals in a moist clayey environment [57]. For ease of understanding, we categorized the κ data into two groups with high κ values indicating deeper soil, distal, channel erosion vs low κ values reflecting topsoil and a proximal provenance (Fig 1; Table 1). These categories are fully concordant with our field observations of the current dynamics of the stream network draining into the lake.

### Sedimentology

**Fig 4:**
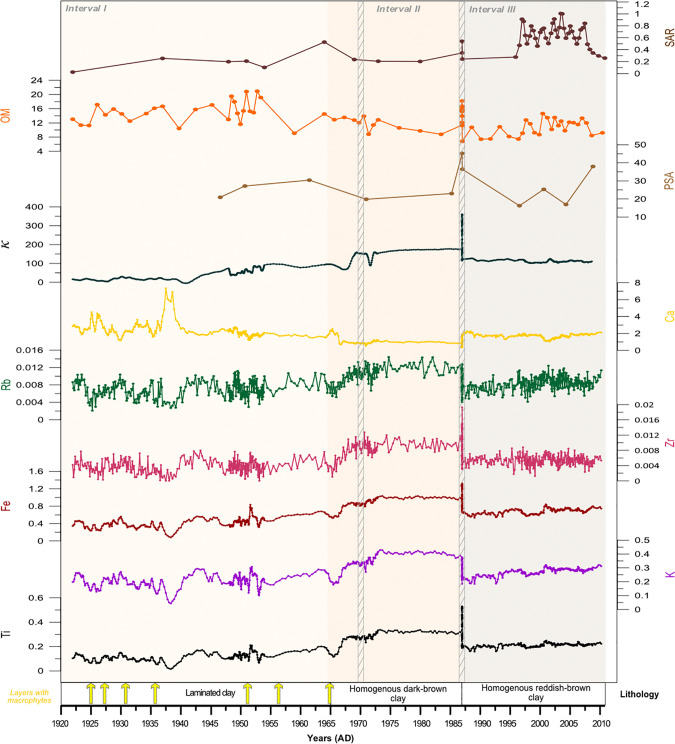
The multi-proxy results of SC-4 core on the age-depth model and showing normalized values for titanium (Ti), iron (Fe), silica (Si), potassium (K), volume magnetic susceptibility (κ, 10^−5^ SI), organic matter (OM %), grain-size (PSA, D50 in μm) and sediment accumulation rates (SAR; g/cm^2^/yr^-1^). Arrows in the lithological description depict layers rich in macrophyte remains. The identified turbidite layers are marked by hatched vertical bars.

Haug et al., (2011) [58] and Smerdon et al., (2017) [59] showed that the geochemical and physical characteristics of lake sediments are reliable proxies for inferring detrital input linked to surface runoff intensity, which in turn is linked to rainfall and/or snowmelt as well as anthropogenic intervention [60]. Here we use Ti, K, Rb and Zr, which show the highest scores in the correlation matrix [see also 18] as indicators of allochthonous sediment input through soil/catchment erosion. As Fe is significantly correlated with Ti and K in the recent sediments, in contrast with trends observed over the last 6000 years at Ighiel [18], it is used here as catchment erosion indicator rather than a redox proxy (Fig 4). The inverse correlations between Ca (Ca-Ti, r^2^ = 0.71) and Si (Si-Ti, r^2^ = 0.49) with Ti points to a predominantly endogenic origin of Ca and Si. The correlation of Ca with Si/Ti (r^2^ = 0.58) suggests that carbonate production has been mediated by biological activity, via for example, algal blooms. The intervals set out below are based on visual lithological identification and variability in geochemical data (S2 Fig).

Between 76–48 cm (spanning 1922–1965*)* in core SC-4, the sediment column is characterized by layers of clayey silts and silty sands as well as thin layers composed of subaquatic macrophytes. The macrophyte-rich layers dated around *~*1925, ~1927, ~1936, ~1951, ~1956 and ~1965 are characterized by marked drops in Ti and K, but increases in OM; they are preceded by white, carbonate-rich layers (also identifiable in the Ca curve) and may point to intervals of low-lake levels driving carbonate precipitation and the accumulation of coarse organic remains.

Titanium, K, Fe, Rb, Zr, κ and OM show variable patterns while SAR remain stable with a slight increase around 1940’s (Fig 4). The detrital elements (Ti, K, Fe) peak around 1930, 1945 and 1953 suggesting an increased allochthonous siliciclastic input which is corroborated by the κ results indicating deeper ravine erosion (Fig 4). In the interval 76–48 cm, an OM content above 15% follows the trend in detrital proxies, pointing to in-wash as the main source of OM. An opposite trend in behavior of the detrital elements is observed for Ca, indicating endogenic carbonate precipitation when low values are registered in Ti, K, Fe and other detrital elements. Low κ values (<30 10^−5^ SI) below 64 cm (1940’s) support this interpretation and point to topsoil erosion of proximal slopes as main source of material into the lake (except for the detrital peaks mentioned above).

The interval 48–25 cm (1960–1980) is characterized by a sharp shift from laminated to massive, homogenous dark-brown, clayey sediments. Titanium, K, Zr, Rb and κ show a sudden increase and suggest accelerated erosional processes and a possible change in the dominant sediment source (Fig 4). The κ values greater than >40 10^−5^ SI suggest an input linked to basal erosion. SAR peaks around 1963 to 0.5 g/cm^2^/yr reflecting a marked increase in sediment flux into the lake (Fig 4). This is corroborated by a decrease in OM (values <15%) and lower Ca suggesting lower productivity and reduced endogenic carbonate precipitation.

The third interval covers the upper 25 cm (spanning 1987–2010) and is characterized by homogenous, reddish-brown clayey sediments (Fig 4), whereas Ti, K, Fe, Zr, Rb and κ values show a steady trend towards slightly lower values, preceding the turbidite. The sediment κ fingerprinting hints at material originating mainly from deeper soil horizons via erosion of the over-deepened ravines. SAR depicts a highly fluctuating trend registering a maximum of 0.9 g/cm^2^/yr only after 1995 and suggesting an unprecedented increase in sediment fluxes.

Given the uniform sediment characteristics of the last 6000 years [18], the presence of significant turbidites in the upper 76 cm of the lake’s profile indicates that flash-floods and/or underwater slope failure has affected Lake Ighiel only very recently. Dating results show that a 6 cm thick turbidite layer identified between 36 and 25 cm in core SC-4 (comprising also very coarse plant remains) was emplaced in 1987 whereas a thinner turbidite, identified solely via diatom assemblages, between 41 and 38 cm, formed in 1970 (S1 Fig). In core SC-3, located in the eastern part of the lake basin near the outflow, two turbidites were also identified; the lower one (at 70–40 cm) emplaced in 1970 is traceable in the sedimentological and dating results, whereas the upper turbidite at 27 cm depth (~2 cm thick) emplaced in 1987, is apparent mainly in the diatom assemblage. The turbidites are characterized by few large celled, motile diatoms, e.g., *Cymatopleura solea*, typical taxa for coarser sediments and turbulent water columns [61]. The appearance of turbidites that are restricted to the upper sediments reflects recent anthropogenic landscape changes, the resulting low catchment buffering and a more responsive system to the impact of rapid hydroclimate events, drastically altering the depositional regime of Lake Ighiel (S4 and S5 Figs). Around the timing of the turbidite deposition, proxy data indicate major lake level declines, followed by enhanced rainfall. The sediment fingerprinting, alongside the poor diatom assemblages typical of coarse detrital input, advocate for deeper erosion with sediments dispatched and transported downslope during torrential event(s), rather than redeposition following slope failure within the basin. This is also suggested by the variable thickness of turbidites and their proximal deposition within the lake at the mouth of the main inflow to the lake basin.

### Diatom assemblages

**Fig 5:**
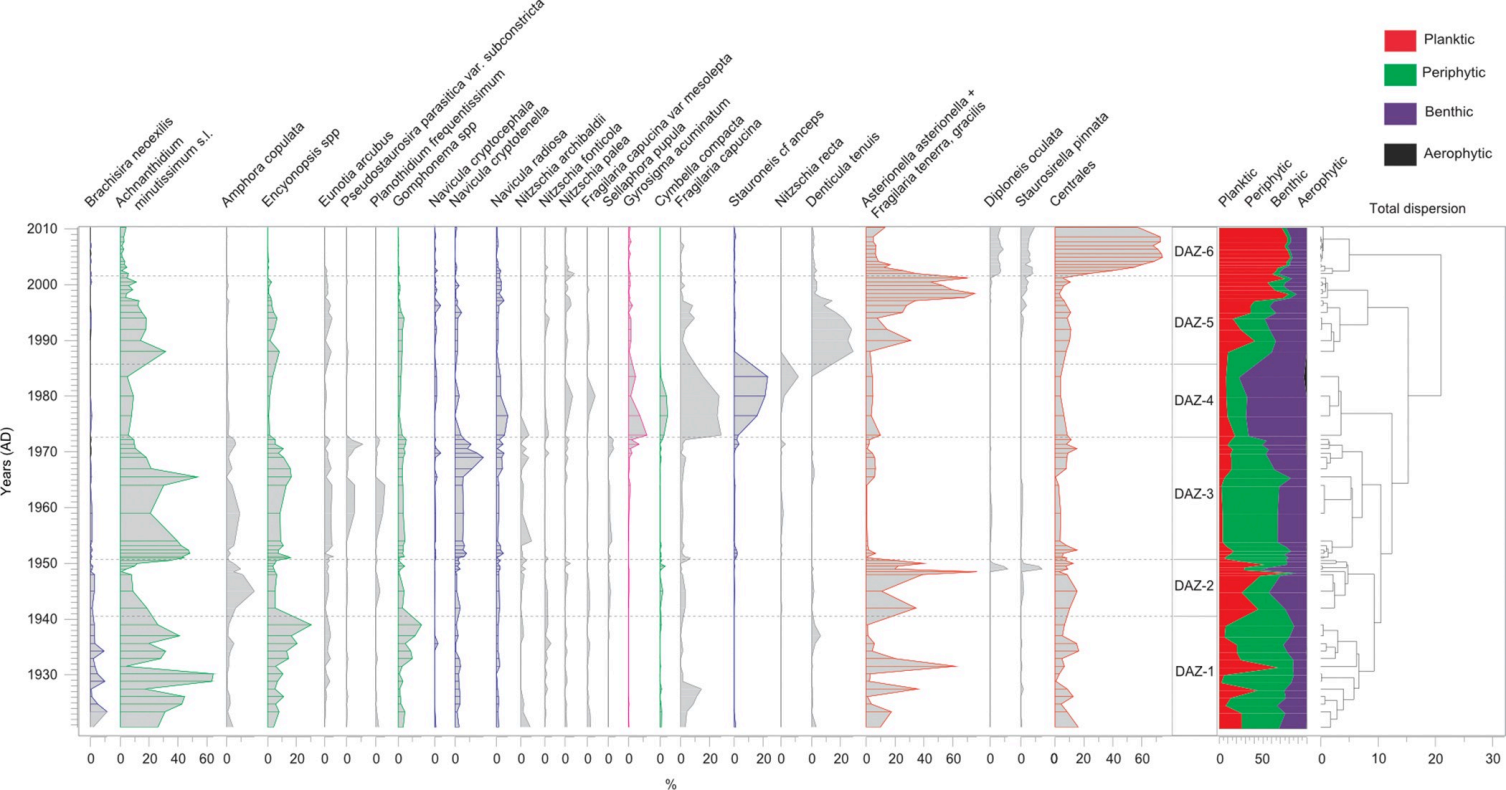
Downcore changes in the relative frequency of selected diatom taxa and the life forms changes in gravity core SC-4 (significant diatom assemblage zones—DAZ as defined by CONISS and validated by the broken stick model [54] are also shown).

In core SC-4, 136 diatom species were identified of which *Achnanthidium minutissimum* s.l., *Asterionella formosa*, *Encyonema* taxa, *Eunotia arcubus* and *Navicula radiosa* are the dominant species (S2 Table). CONISS analysis identified six significant diatom assemblage zones labelled here IGH-SC4-DAZ-1 to 6 (Fig 5).

IGH-SC4-DAZ-1 (77–62 cm; 1920–1941) is dominated by small periphytic taxa such as *Achnanthidium*, *Encyonopsis* and *Gomphonema* species. *Asterionella formosa* shows large fluctuations while *Brachysira neoexilis*, *Eunotia arcubus*, *Navicula cryptotenella* are common. *Brachysira neoexilis* is a good indicator of oligotrophic and mesotrophic habitats [37], whereas an alfa diversity (expressed by the number of taxa) at 24.5±3.8 suggests a relatively stable, moderately productive environment (Fig 5).

IGH-SC4-DAZ-2 (62–55 cm; 1941–1951) is clearly dominated by planktic taxa such as *Asterionella formosa* and centric diatoms. *Amphora copulata*, a species common in mesotrophic to polytrophic habitats, shows a peak. Other taxa identified have very low relative abundances. This zone shows a higher diatom diversity, the average number of taxa is 30.9±4.2 and the increasing abundance of planktic diatoms may indicate increasing nutrient availability in the water column and/or an increasingly pelagic habitat and thus, greater water depths.

IGH-SC4-DAZ-3 (55–42 cm; 1951–1972) is dominated by *Achnanthidium minutissimum* s.l., but *Encyonopsis* taxa (mainly *E*. *cesatii*, *E*. *minuta E*. *subminuta*) are also common. It is worth mentioning that *E*. *cesatii* is an indicator of good/high ecological status [37, 62]. *Eunotia arcubus* is highly abundant in this zone. Since this species is typical for carbonate-rich, oligo-mesotrophic lakes and prefers low-light environments in stagnant waters [37], its abundance suggests stable environmental conditions with inter-species competition maintaining a rich diatom flora. *Planothidium frequentissimum*, *Pseudostaurosira parasitica* var. *subconstricta*, *Amphora copulata*, *Navicula cryptotenella* and *N*. *radiosa*, diatom taxa with broad tolerance and wide ecological amplitude, are also common. The number of taxa is the highest in this zone at 32.4 ± 6.6. Taken together, this diverse diatom assemblage and the lack of planktic taxa, indicate a shallower lake with dense macrophyte vegetation.

In zone IGH-SC4-DAZ-4 (42–20 cm, 1972–1993) the diatom assemblage is dominated by *Fragilaria capucina* and *Stauroneis* cf. *anceps*. The species with the highest indicator value is *Gyrosigma acuminatum*. As it is tolerant of river-borne sediment influx, this species is commonly used to identify intervals of river flooding [63]. In our record, the occurrence of *Gyrosigma acuminatum* (>5%) is consistent with trends in the geochemical elements denoting detrital input. Also, the aerophytic diatoms that appear in this zone support this interference, clearly documenting slope or river in-wash consistent with evidence from the erosion proxies (Fig 5). The average number of taxa significantly decreases, reaching only 23.6±4.2.

IGH-SC4-DAZ-5 (20–12 cm; 1993–2001) is dominated by planktic diatom *Asterionella formosa*, reaching more than 60% in the upper part of the zone, while the abundance of *Achnanthidium minutissimum* s.l. gradually decreases. The number of taxa fluctuates, but the average is rather low (29 ± 6.3). *Denticula tenuis*, a diatom specific for alkaline lakes and frequently found in the littoral area of lakes and in running waters, registers peak abundance in this zone. Overall, these changes point to higher phytoplankton productivity, alongside a significant alteration of the whole diatom community. This ecological shift cannot be explained solely by lake level changes and/or modification of the available nutrient budget. It most likely reflects a complex interaction of environmental drivers that resulted in significant changes in the algal community.

Zone IGH-SC4-DAZ-6 (12–0 cm, 2001–2010) exhibits the most substantial shift in the diatom assemblage with the decrease in the abundance of *Asterionella formosa* and an increase of small celled centric taxa (mainly *Pantocsekiella costei* with *P*. *delicatula*). Besides planktic centric, and also planktic *Asterionella formosa*, the benthic *Diploneis oculate*, known from carbonate rich freshwater habitats with moderate electrolyte content [37], and the widespread *Staurosirella pinnata* also register more than 5% relative abundance. The alfa diversity of this zone is low with an average of 21.5 ± 2.9. These data point to accelerated shifts in diatom assemblages for the last decade alongside an increase in euplanktic productivity.

#### Ordination of diatom assemblages

**Fig 6:**
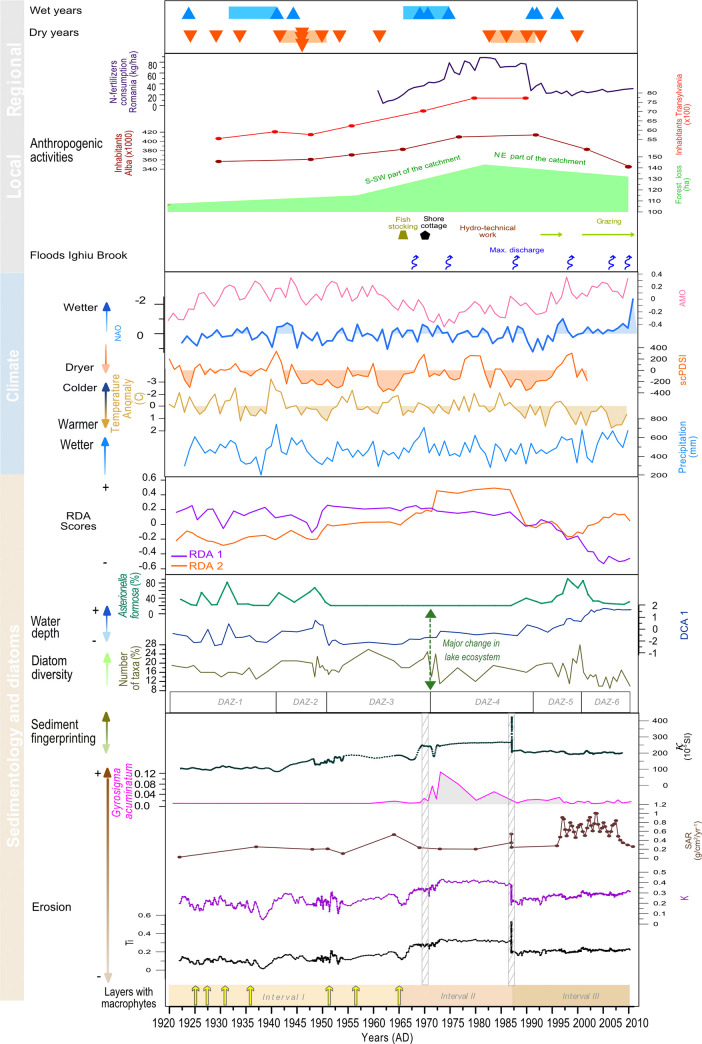
Selected proxies from gravity core SC-4 from Lake Ighiel showing the main lithological intervals, the organic-rich layers with subaquatic macrophytes, normalized Ti and K, sediment accumulation rate, magnetic susceptibility used for tracing sediment sources and diatom—erosion related species (*Gyrosigma acuminatum*), number of taxa, DCA 1 (Note: see text for curve interpretation over the last two decades) and changes in diatom species *Asterionella formosa*. The climate data includes mean annual precipitation and annual temperature anomaly from the Cluj-Napoca meteorological station [45, 46], dryness (annual mean scPDSI index) [47, 48] and NAO and AMO indices [49, 50]. The upper two panels mark the main natural hydroclimate events (floods) alongside identified anthropogenic activities (e.g., land-use, number of inhabitants, N-fertilizers use) that affected the lake catchment and its surroundings over last decades. Regionally documented wet and dry years are marked with triangles [69, 70]. The turbidites are marked with grey vertical bars.

The sample scores of DCA-axis 1 are plotted on the age-depth model results in Fig 6. The high sample score values on DCA-axis 1 explain 34.6% of the variance and are associated with *Asterionella formosa*, centric taxa (e.g., *Pantocsekiella costei*, *P*. *delicatula*), as well as the benthic *Staurosirella pinnata* and *Diploneis oculata*. Based on the associated diatom life forms and habitat preferring taxa, DCA-axis 1 can be interpreted as reflecting lake-level changes. However, the higher planktic ratio in the upper part of the profile may possibly reflect changes in trophic and/or thermal conditions and not necessarily only lake-level variations. DCA-axis 2 explains 13.6% of the variance and high scores were associated with *Asterionella formosa*, *Navicula radiosa*, *Eunotia arcubus*, *Denticula tenuis*, while low sample scores were associated with centric diatoms and benthic *Diploneis oculata*, *Stauroneis* cf. *anceps*, *Nitzschia recta* and *N*. *archibaldii*. The interpretation of DCA-axis 2 is not straightforward; further investigations on the auto-ecology of dominant diatoms (mainly the centric diatoms that represent at least ten different taxa; Ács (unpublished data)) are required.

### The relationship between diatom assemblages and environmental proxies

**Fig 7:**
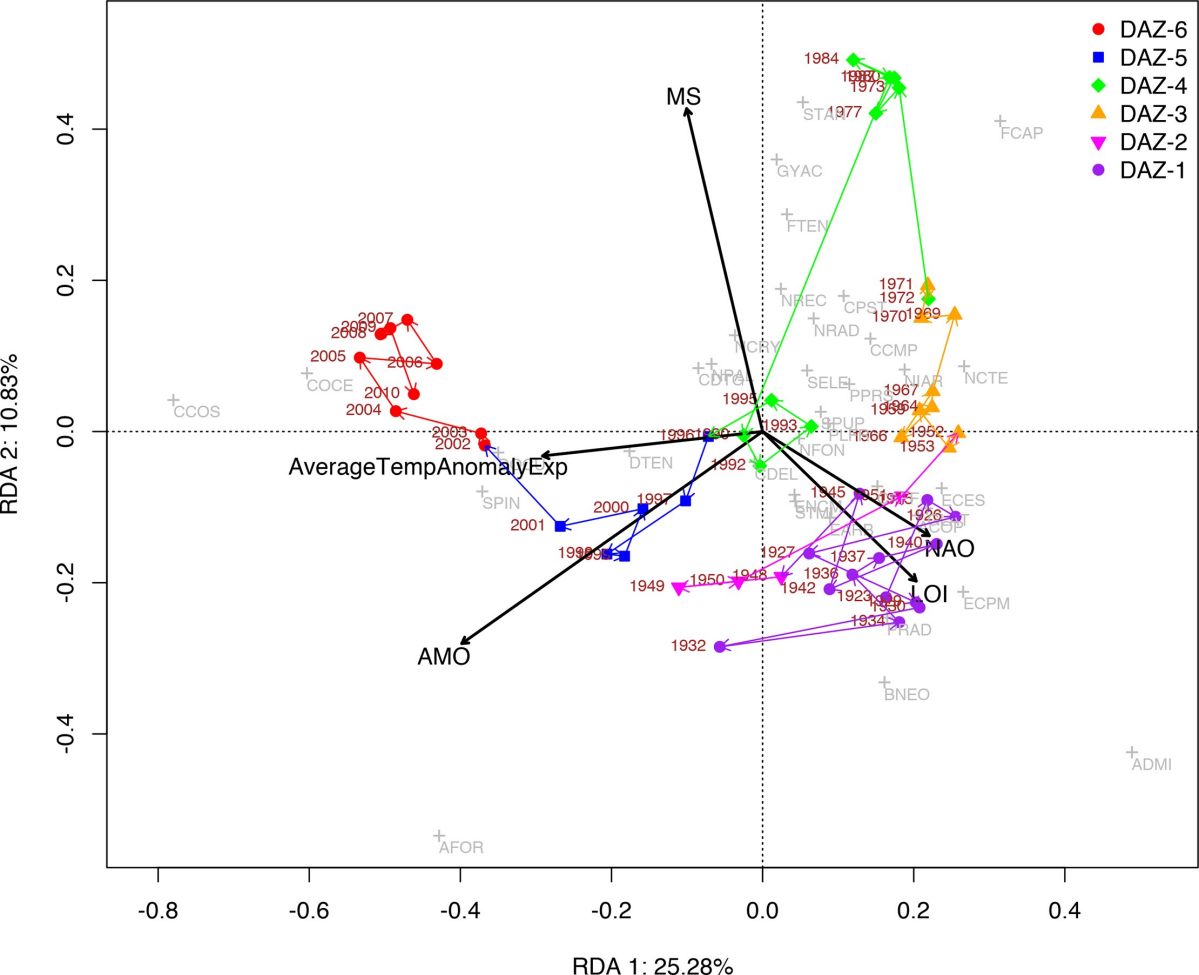
Redundancy analysis (RDA) biplot showing the significant correlations between diatom assemblages and explanatory environmental variables such as sediment properties (K magnetic susceptibility, OM-LOI 550 organic matter) and climate parameters (Average Temperature AnomalyExp, AMO, NAO). Abbreviated names for the main diatom taxa are displayed in grey (for full species name consult S2 Table). The lines show the temporal trajectories.

RDA analysis was used to investigate the relationship between the diatom assemblages (response variable), sediment proxies and hydroclimate forcing variables. RDA was run on the entire SC-4 profile (excluding the turbidite). The results show that shifts in the diatom assemblages could be largely explained by concurrent changes in sedimentological proxies such as OM and κ and/or hydroclimate variables (NAO, AMO, Temperature Anomaly). RDA Axis 1 explains 25.28% of the total variance and is positively correlated with small periphytic diatom taxa, i.e. *Achnanthidium* and *Encyonopsis*, *Fragilaria capucina* and *Navicula cryptotenella*, OM and NAO. This correspondence appears significant for the interval with the laminated sediments spanning between 76 and 48 cm sediment depth (1920–1950). The negative direction of RDA Axis 1 shows high correlation with the planktic taxa, i.e., *Asterionella formosa* and centric *Pantocsekiella costei* and *Pantocsekiella ocellata* as well as benthic species, *Staurosirella pinnata* and *Diploneis oculata*. For the upper part of the sediment profile spanning 12 to 0 cm (2001–2010), the most significant environmental explanatory variables are AMO, Temperature Anomaly and κ (Fig 7).

RDA Axis 2 explains 10.83% of the variance and it is positively correlated with benthic taxa, such as *Stauroneis anceps*, but also *Fragilaria capucina* and *Gyrosigma acuminatum* (erosion indicator) and κ with a stronger correlation for the interval 1970 to 1980. The negative direction of Axis 2 is correlated with the planktic taxa *Asterionella formosa* and small-celled periphytic diatoms such as *Achnanthidium minutissimum* and *Brachysira neoexilis*, and the environmental variables AMO and OM that are representative for the bottom part of the record (1920–1965) where the sediments are laminated and macrophyte layers are present (Fig 7).

### Land-use changes

Land-use changes were calculated as forest loss percentages (%) for each time step relative to the initial extent of the forest (i.e., year 1870). Therefore, when compared with the initial forest, forest loss increased to 4% in 1957 with forest loss predominantly in the eastern and central part of the catchment (Fig 3). In 1982 forest loss reached 7% (compared to 1870) with losses mainly in the south-western part of the catchment (Fig 3). In 2012 forest loss showed an increase of 6% (compared to 1870), slightly lower when compared with the preceding period, and mainly affecting the eastern and central-western part of the catchment (Fig 3).

## Discussion

### Proxy responses to environmental changes between 1922 and 1964

During this interval the biotic and abiotic indicators reflect important changes in both the catchment and lake ecosystem. The main proxies for detrital input, including terrigenous elements (Ti, K, Zr) and magnetic parameters, i.e., κ, are characterized by variable patterns and peak around ~1930, ~1945 and ~1953 pointing to topsoil erosion as the main contributor of allochthonous material to the lake (except the detrital peaks that in turn likely reflect sudden short-term inputs from deep channel erosion) (Fig 6). This inference is supported by land-use map-based analysis (Fig 3) documenting no significant changes in forest cover in the catchment of Lake Ighiel and thus limited slope destabilization and deeper soil erosion. It is more likely that the sediment source during this interval was mainly topsoil from nearby slopes. However, between the 1940’s and 1960’s the detrital proxies register a significant increase and, alongside the sediment fingerprinting data (Fig 6), might document a source shift linked to stronger inputs from deeper soil erosion via the ravines.

In terms of the lake ecosystem, the diatom assemblages within IGH-SC4-DAZ-1 to 3 (1920–1972) point to an oligo-mesotrophic water column with no major disturbances of the lake’s ecosystem. The diatom-based lake level reconstruction (DCA-1) [64] and other changes in the diatom assemblages indicate shifts from high lake levels and increased trophic state around ~1933, ~1940–1950 to low lake levels and low trophic state around ~1928–1930, ~1939 and ~1951–1965 (Fig 6).

In addition, thin layers composed of sub-aquatic macrophytes were deposited around *~*1925, ~1927, ~1936, ~1951, ~1956 and ~1965 (Fig 6). The different sampling resolution for the abiotic (1 mm) and biotic proxies (1 cm) must be taken into consideration; however, at around the same timing the diatoms show a high ratio (<70%) of periphytic algae, with a marked abundance of *Achnanthidium minutissimum*, and lack of planktic diatoms (Fig 5). *Achnanthidium minutissimum* typically grows attached to aquatic plants and has a higher capacity to adapt to environmental changes. Therefore, its presence points to extensive aquatic vegetation and low lake levels [65, 66]. Specifically, the macrophytes and the periphytic algae that live attached to them grow rapidly when water levels decrease and other limiting conditions are met, especially during the growing season (e.g., calm wind conditions, temperature increase and nutrient availability) [67]. Several studies have shown that temporary declines in water-level may enhance macrophyte abundance [67]. Taken together, the presence of layers with macrophyte and the presence of periphytic algae probably indicate seasonal (negative) shifts in the local hydrological balance, i.e., low lake level during dry intervals [67, 68]. This hypothesis linking macrophyte layer deposition with dry periods is also supported by changes in precipitation trends showing decreased precipitation amount and scPDSI (Fig 6), portraying increased dryness during the deposition of macrophyte layers and thus, might reflect the impact of regionally documented excessively dry periods on our record, i.e. at *~*1925, ~1927, ~1936, ~1951, ~1956 and ~1965 [69–75] (Fig 6).

Notwithstanding the enhanced detrital input as shown by the geochemical and magnetic parameters, the diatom-inferred high lake level stands identified at ~1930, ~1945 and ~1953 correlate well with regionally wetter conditions as indicated by the higher values in mean monthly precipitation (Fig 6) showing higher erosion under precipitation events.

Moreover, the ordination analysis (Fig 7) shows that, for this first interval, changes in diatom assemblages are linked to changes in organic matter, probably reflecting nutrient availability/delivery and overall productivity, and also with the NAO that acts as large-scale precipitation modulator over the area, influencing decadal variability in rainfall distribution [76]. Nonetheless, AMO/AMV, a temperature decadal mechanism linked to the occurence of extreme summer-autumn warm events [50, 73, 77], may also be connected to proxy changes in Lake Ighiel. For example, higher temperatures likely promoted macrophyte growth and changes in hydrological balance (i.e., during dry periods) (Fig 7). Taken together, the documented lake ecosystem changes align well with the reconstructed catchment changes suggesting a common mechanism, i.e., most likely natural climate variability expressed as changes in temperature and precipitation, driving the response of both abiotic and biotic proxies.

Furthermore, the map-based land-use analysis does not show significant landscape changes, which is also supported by the area’s low population suggesting an overall modest anthropogenic impact. Such minor changes in land-cover are also observed in other mid- and high-altitude mountain lakes across the Carpathians [25, 78]. Such records suggest that between the 1920’s and 1960’s the responses of the paleolimnological proxies at Lake Ighiel closely track natural hydroclimate variability in parallel with other examples from the region [25, 78].

### Proxy variability between 1964 and 1987

From the 1960’s the depositional regime experienced a marked shift from the laminated silty clays that characterized the preceding 6000 years [18] to massive, homogenous sediments. In line with this lithological change (at 48 cm) Ti, K, Fe, Rb, Zr and κ show a sudden shift to consistently higher values, likely reflecting sediment input from enhanced basal soil erosion (Fig 6). A change in the diatom assemblage (IGH-SC4-DAZ-4) starting gradually around 1965 with a decrease in periphytic forms and an increase in planktic diatoms is documented (Fig 6). There is a marked boundary around 1971, but it remains unclear whether this boundary reflects a tipping point in the lake’s ecosystem. Tipping points, that mark the shift between contrasting system states occur when external conditions reach thresholds that trigger an accelerating transition to a contrasting new state [79]. Nevertheless, the presence of *Gyrosigma acuminatum*, detected exclusively in this interval, an erosion-related diatom alongside other aerophytic diatoms appears consistent with the shift in geochemical and magnetic proxies documenting exceptional allochthonous inputs at certain intervals into the lake and thus, enhanced erosional activity (Fig 6). The diatom-based lake-level reconstruction (DCA-1) does not depict major changes in lake stands, only a minor increase (Fig 6).

The two-fold increase in detrital proxy levels, followed by a smoother trend, corroborated by trends in organic matter, SAR and changes in diatom assemblages provide evidence for a major disequilibrium in the catchment and lake ecosystem at certain time intervals, i.e., 1970 (Fig 6). The map-based forest cover estimation (reference 1982) clearly shows extensive forest loss over the southern and south-western part of the catchment and suggests slope destabilization under forestry-driven activities (Fig 3). This increase of human pressure is further supported by evidence of population growth both locally and regionally (Fig 6). During the same time interval, official documents [80] indicate a significant anthropogenic impact through road construction, hydro-technical works and mechanized timber exploitation undertaken in the proximity of the lake.

It is worth noting, however, that this interval also coincides with an increase in precipitation trend and scPDSI that led to regional floods (as reported in Ighiu town hall documents [80]) marking the inception of a wetter period [69, 70]. As such, it is very likely that landscape destabilization through forestry and other human activities in the area rendered the steep catchment slopes even more susceptible to enhanced erosion under predominantly wetter than average conditions.

The RDA results support the assumption that over this interval changes in the composition of diatom assemblages were connected to a greater input of siliciclastic material and thus major catchment disturbance through deforestation (Figs 3 and 6). A ca. 5-year time lag is observed between major change in the aquatic ecosystem (IGH-SC4-DAZ-4) and sedimentology (Interval II), which suggests a slightly delayed response of the diatom community to catchment disturbance. However, this is not surprising given that diatoms like other biological communities can show resilience time/hysteresis in response to a new stressor [81]. Furthermore, from 1966 the stocking of the lake with fish (*Salmo trutta fario*, *Oncorhynchus mykiss*, *Hucho hucho*, *Phoxinus phoxinus*, *Leuciscus cephalus*, *Cottus gobio)* [29] may have contributed to some of the changes in the lake ecosystem. Fish stocking is a serious anthropogenic perturbation to natural aquatic ecosystems as it can fundamentally alter nutrient cycles and stimulate primary production by accessing benthic phosphorus sources that are not normally available to pelagic communities in oligotrophic mountain lakes [82, 83]. Such changes were observed in, for example, Lake Opeongo, Canada [82].

### Proxy responses between 1987 and 2012

From 1987 the responses in lake proxies are remarkable; the geochemical data indicate sustained erosional activity, with SAR reaching unprecedented values denoting enhanced sediment mobilization, especially over the past two decades (Fig 6). The abrupt variability observed in the diatom assemblages appears in concert with the sedimentological data and exhibit the most substantial shift with open water planktic species almost completely replacing the benthic taxa (Figs 5 and 6). This prominent change in the diatom community marks the inception of meso-eutrophic conditions and was likely driven by higher nutrient availability [84–86]. The change towards a planktic-dominated diatom community might hint at habitat disturbance for the benthic taxa, heralding a marked transformation in the lake ecosystem during last decades. Our interpretation of significant recent eutrophication is also supported by the results of [29].

In this interval further hydro-technical works to stabilize the catchment’s river network (southern channel) took place and there was a dramatic expansion in local sheep farming [80]. Although sheep and cattle grazing in the Carpathians is a traditional activity [22], in the surroundings of Lake Ighiel, a 43*%* increase in sheep numbers was reported between 1993–1996 and between 2003–2010 [43, 80]. As recent grazing activities in the wider Apuseni Mts have been shown to have significantly impacted the landscape [87] through changes induced in the plant community and soil stability, this exceptional increase in live-stock might be an important factor (although not the sole one) in explaining the responses observed in our paleolimnological proxies.

The forest loss estimation [41] shows localized forest loss mainly in the east of the catchment, along the Striglau valley (Fig 3). However, when compared with previous estimates (reference dates 1957 and 1982), recent forest loss is less extensive (Fig 3). Furthermore, as the digital elevation model shows (Fig 1), the eastern catchment where most recent forest loss occurred exhibits greater slope angles. It might be expected that landscape changes in this part of the catchment will have a marked impact on Lake Ighiel’s sedimentation regime by enhancing the sediment input. It is well documented that forest disturbances (loss of forest cover), by removing vegetation cover and exposing the soil to direct rain impact, activate geomorphological processes and promote sedimentation especially on steep slopes [78, 88, 89]. Thus, deforestation on such steep slopes, in addition to grazing intensification, might have contributed to the high but variable SAR, indicative of enhanced intra-catchment variability in sediment availability. Furthermore, the meso-eutrophic lake status as reconstructed from diatoms might be interpreted as an anthropogenic signal driven by increasing nutrient supply directly linked to enhanced grazing activities in the area. Overall, it appears that recent anthropogenic activities are linked to increases in the sediment accumulation rate that, in turn, impacted on the lake’s ecological status.

However, results from multivariate analysis (RDA axis 1 in negative direction) also show that changes in planktic centric taxa over the last decades may also reflect to some extent hydroclimate changes, such as temperature variability and AMO (Fig 7). Such a connection between changes in diatom communities and hydroclimatic parameters is not surprising given that a temperature increase alongside other factors like water-column turbidity, nutrient availability, ice-cover dynamics may induce a cascade effect on the biological community, e.g., the biological productivity is accelerated when temperatures increase [86, 90–93]. Thus, it is reasonable to assume that during the last two decades hydroclimate variability might exacerbate the impact of the well-documented anthropogenic activities on the Lake Ighiel ecosystem, further threatening the stability of this fragile mid-altitude ecosystem (Fig 6).

### Nitrogen (N) as a potential driver of recent ecosystem change in Lake Ighiel?

In the Northern Hemisphere mountain lakes, and to some extent all over the world, it is well recognized that higher rates of atmospheric N deposition and/or N catchment export coincide with substantial changes in algal communities triggering ecosystem turnover [94–97]. *Asterionella formosa* is often regarded as indicator of moderate N-enrichment in oligotrophic alpine lakes [98] and the proliferation of *A*. *formosa* correlates with nutrient enrichment, especially when atmospheric N deposition intensifies. This species is known as a common and often dominant planktic diatom in mesotrophic and eutrophic lakes worldwide, but more recently its abundance has also increased in oligotrophic lakes [99].

The relationship between the abundance of *Asterionella formosa* and N deposition is a hot topic in ecology and paleolimnology; given that practically no information is available from the Carpathian lakes, we analyzed this question with special attention. In our model interrogating the key drivers of diatom community change in Lake Ighiel (Fig 6), forcing from N deposition could not be explored due to the shorter period of the dataset, and lack of direct data on N emission/deposition. Therefore, we used the N fertilizers consumption in Romania as a rough estimation of anthropogenic N deposition [100] to explore if/how the Lake Ighiel ecosystem might have responded by analyzing changes in *Asterionella formosa* and making a comparison with responses in other diatom records.

It is well known that the increased availability of reactive N over the past century as a product of industrialization and agricultural intensification has increased primary production in mountain lakes [101]. In Romania, N fertilizer consumption also increased in the second half the 20^th^ century showing a clear peak between 1975 and 1990 (Fig 6). After the political changes in 1989 that led to the collapse of the state-planned economy, fertilizer use fell drastically while soil N input halved within one year [101] (Fig 6).

Surprisingly, the rise of *Asterionella formosa* in the diatom assemblages of Lake Ighiel began at the time of decreasing use of N fertilizers in Romania in the 1990’s (Fig 6). To explore the response of the ecosystem to N deposition, we calculated Pearson correlation between the relative abundance of *Asterionella formosa* and data on the annual N fertilizer use in Romania available for the interval 1961 and 2014 [101]. The correlation coefficient indicates a slightly negative, marginally significant correlation (r = -0.35, p = 0.052). Given the complexity of N deposition in the environment with limiting factors such as the N:P ratio and the complex ecological responses of lakes to post-industrial environmental changes [95], in light of available data the recent responses of Lake Ighiel ecosystem cannot solely be attributed to N deposition; rather they reflect a combination of drivers among which erosion following land-use changes stands-out.

The complexity of responses in diatom records has been demonstrated by the opposing trend observed in several lakes in western USA that document a decline of centric diatoms coinciding with a successive increase in planktic araphids (mainly *Asterionella formosa* with *Fragilaria tenera* group and *F*. *crotonensis*). Lines of evidence [e.g., 94–96, 99] highlight that turnover in ecosystems is caused by the combined effect of increased nutrient availability and deposition following post-industrial pollution as well as global warming temperatures. At Lake Ighiel local drivers, such as proximal land-use changes and fish stocking can also be added.

### Local anthropogenic signals vs regional climate change in the Ighiel record

Natural climate variability and anthropogenic activities have shaped Ighiel’s ecosystem and limnological responses for millennia. The key aim of our work is to disentangle local, anthropogenic drivers, documented via land-use map-based analysis, from regional climate changes, inferred through precipitation, temperature, drought indicators (scPDSI) and their main driving mechanisms (NAO, AMO), via Lake Ighiel’s sedimentary record over the last nine decades. Previous work on this record showed that on a long-term timeframe (i.e., the last 6000 years) sedimentological data closely track rainfall variability induced by large-scale atmospheric teleconnections, e.g. NAO [18]. From a long-term perspective, in the Ighiel record and in other sites across the globe [1, 12], the main drivers of recent changes in paleolimnological proxies are mainly related to anthropogenic activities, e.g., forestry, urbanization, resource exploitation. Nonetheless, high-resolution investigations can still identify forcing through natural hydroclimate variability.

The first noticeable changes spanning the interval between 1920–1960 were decreased erosional activity, deposition of macrophyte layers and low lake levels. These were coincident with regional dry periods. The RDA analysis showed a clear response in the lake proxies to changes in hydroclimate (dry vs wet periods) driven by large-scale teleconnections, AMO/NAO. This interference of climate-driven lake changes was also supported by the occurrence of only minor changes in land-use as shown by the map-based analysis; modest human impact over this period was also observed regionally, in other mountain records [25, 78].

Starting from 1960, the sedimentation regime began to change; the accumulated sediments turned homogenous and the geochemical parameters marked a shift towards high intensity catchment erosion, while the diatom assemblages pointed to a shift in the lake’s ecology. These changes seem to have been driven by anthropogenic activities including catchment forest clearance and landscape destabilization, registering a higher loss (7% relative to 1870) when large parts of the southwestern catchment were turned into pastureland. Local fishery seems to have affected the diatom community. These changes coincided with an increase in regional population numbers and also appear to have put greater pressure on natural resources, with timber felling as an important economic activity over large areas in CE Europe [26]. Regionally, similar environmental responses were found in other lowland and mid-elevation sites [25] and also reservoirs showed increased silting [102]. Remote alpine and arctic sites also showed indications of intense human impact [103] and together mark a rather global, stronger anthropogenic imprint on sediment responses.

Over the last four decades, forest loss remained at high levels and areas closer to the lake were deforested, grazing intensified causing a sharp change in erosional activity and the lake’s ecosystem. Overall, anthropogenic activities overprinted the natural hydroclimate variability. Alpine lakes from the Carpathians [25], North America and also arctic sites show similar changes and highlight the anthropogenic dominance of recent environmental changes [103] as reflected in our lake sediment record.

### Implications for restoration targets

Paleolimnological assessments provide useful toolkits in developing effective management and conservation strategies for lake ecosystems under threat [93, 104–106], but such endeavors in central-eastern Europe remain limited. Although it is rather unrealistic to target the return of Lake Ighiel’s ecosystem to the baseline conditions [18] prior to the major human disturbances of the last decades [90], our results provide crucial information about the current trajectories of change. Our data indicates that over the past century both natural hydroclimate variability and human activities have acted synergistically as the main drivers of change at Lake Ighiel, clearly highlighting that its ecological integrity is at risk (Fig 6). This risk might be exacerbated under the projected (and expected) changing hydroclimate regime in the wider area (extreme events, aridification) and increasing human pressure [18, 25]. Action measures must be undertaken to prevent a complete overturning in its ecological status, especially as Lake Ighiel is a protected area of national interest (IV Category, IUCN), although these legal prerequisites are generally ignored with proximal lake-catchment degradation evident (S5 Fig). To limit the risks associated with such degradation, a restoration scheme must be employed and oriented towards limiting the eutrophication process. Restoration targets must include better land-use management designed to restrict deforestation and grazing activities, limit deep-soil erosion, especially on steeper slopes and along stream channels controlling sediment and nutrient input to the lake via runoff. These are the major factors influencing lake sedimentation and the onset of eutrophication documented in our study.

## Conclusions

Our reconstruction offers an integrated paleolimnological perspective on the type of changes that lake-catchment system at Ighiel has faced over the past nine decades. Our integrated, multi-proxy approach based on sedimentological and diatom analyses, combined with data from local archives and cartographic resources, has allowed the reconstruction of recent environmental and land-use changes in Lake Ighiel’s catchment highlighting the main factors driving them. The analyses of catchment soil samples reinforced our interpretation of sediment sources and pathways allowing for an appraisal of process/activity-based changes in the catchment and the subsequent responses in the paleolimnological proxies. We show that for the most recent decades in the development of Lake Ighiel, the interplay between hydroclimate variability and increased anthropogenic pressure through landscape changes induced significant shifts in sediment availability and lake ecology as follows:

- on a short decadal scale, spanning the 1920 to 1960, the paleolimnological proxies sensitively record changes in hydroclimatic conditions; this is shown by the deposition of organic layers with macrophytes coupled with rapid changes in diatom assemblages with dominant periphytic taxa closely tracing significant lake level drops in dry years;
- by the 1960, intensified human activities, mostly by mechanized timber felling and fishery development, suddenly altered the sedimentation regime, sediments became homogenous and geochemical parameters show high and steady erosion patterns, while diatom assemblages show a marked shift in lake ecological status;
- over the last four decades, changes in sediment deposition together with the marked erosional pattern documented by our proxies (three fold higher than previously registered), show that geomorphological thresholds were crossed, including the formation of deep-ravines draining towards the lake (that prompted hydrotechnical works to be undertaken to stabilize the exposed slopes). Forest loss continued and grazing intensified significantly; the abrupt, sharp and unprecedented changes observed in diatom assemblages directly hint at a highly human-impacted landscape driving ecological changes;
- we include a first identification of the possible effect of nitrogen fertilizer in Romania (as N deposition evidence) on a lacustrine diatom community. At Lake Ighiel, however, the impact of an N increase on the aquatic ecosystem was relatively weak and masked by the other drivers of environmental change at this site.

Our study shows that over the most recent decades, Lake Ighiel provides an exceptional record for disentangling the interplay between hydroclimate variability and increased anthropogenic activity in the mid-altitude Carpathian area. We demonstrate that the observed ecological responses can be satisfactorily explained when considering a combination of natural and anthropogenic, local and regional drivers of change. The connection between the factors driving changes in the Ighiel catchment, and the subsequent responses in lake proxies, are multi-faceted. They highlight the vulnerability of mid-altitude environments, particularly in central-eastern Europe, to recent anthropogenic pressure and climate change. High-resolution palaeoenvironmental studies from this interesting and understudied region appear essential for further disentangling the drivers of the recent environmental change in the Carpathians and informing conservation and restoration planning.

## Supporting information

S1 Fig Correlation of short gravity cores SC-3 and SC-4 based on volume magnetic susceptibility (κ, 10^−5^ SI). The hatched vertical bars mark the turbidite layers.
(TIFF)

S2 Fig Core SC-4 line-scan displaying the normalized titanium (Ti) curve, visible organic layers with subaquatic macrophytes and the corresponding ^210^Pb ages. (TIFF)

S3 Fig Description of the five soil profiles taken from lake Ighiel catchment. The location and picture of the soil profile is shown on the left side while the physical characteristics (composition, color), soil horizons and magnetic susceptibility is presented on the right side of each figure. Please note that soil type identification is based mainly on qualitative indices following their description following the national soil map.
(TIFF)

S4 Fig Drastic seasonal changes in the Ighiel’s water level as seen in autumn (November 2014, left photo) and summer (July 2019, centre and right photo). Photos taken from the north-western side of the lake in the Plesanului valley (photo courtesy of Daniel Veres and Aritina Haliuc).
(TIFF)

S5 Fig Hydrotechnical works in the southern part of the Ighiel catchment with felled trees and branches to the left along the forest road (July 2019, photo courtesy of Aritina Haliuc). (TIFF)

S1 Text Description of soil profiles from the Ighiel catchment. (DOCX)

S1 Table The CRS model used to calculate the age and the sedimentation rate for core SC-4. (DOCX)

S2 Table The most abundant diatoms in IGH-SC4 core. The taxonomical position of the diatoms was confirmed with the Algaebase [1]. The abbreviated name of the main taxa used in Fig 7 (main text) after [2].
(DOCX)
