# Correction: Climate and land-use as the main drivers of recent environmental change in a mid-altitude mountain lake, Romanian Carpathians

**DOI:** 10.1371/journal.pone.0248253

**Published:** 2021-03-02

**Authors:** Aritina Haliuc, Krisztina Buczkó, Simon M. Hutchinson, Éva Ács, Enikő K. Magyari, Janos Korponai, Robert-Csaba Begy, Daniela Vasilache, Michal Zak, Daniel Veres

An additional affiliation is missing for the sixth author. Janos Korponai is also affiliated with Department of Water Supply and Sewerage, Faculty of Water Sciences, University of Public Service, Baja, Hungary.
