# Supplementary figures and images for: Climate and land-use as the main drivers of recent environmental change in a mid-altitude mountain lake, Romanian Carpathians
Source: PLoS One. 2020 Oct 1;15(10):e0239209. doi: 10.1371/journal.pone.0239209 (PMC7529234; doi:10.1371/journal.pone.0239209)

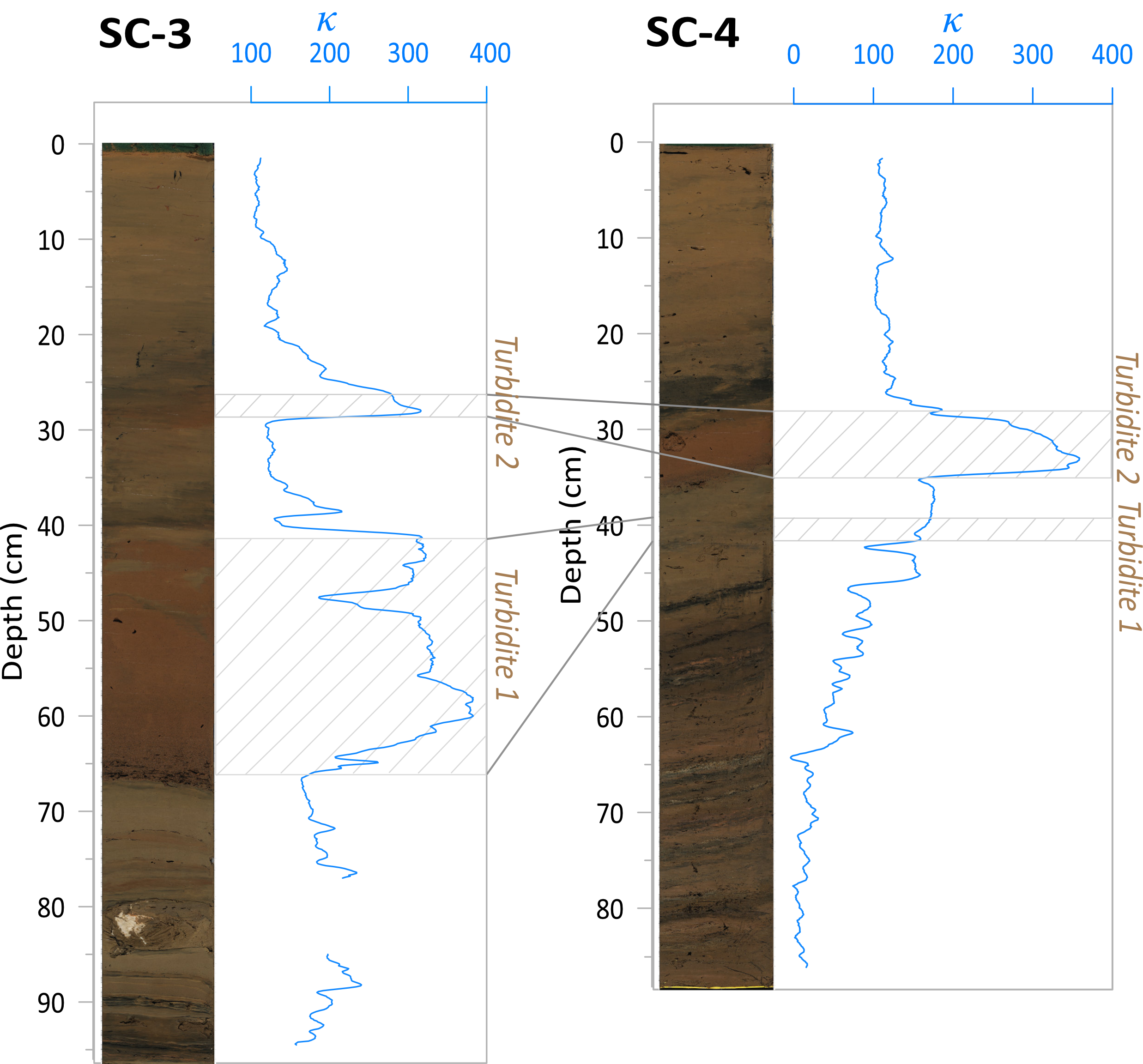

Supplement: S1 Fig — The hatched vertical bars mark the turbidite layers. (TIFF) [file pone.0239209.s001.tiff]

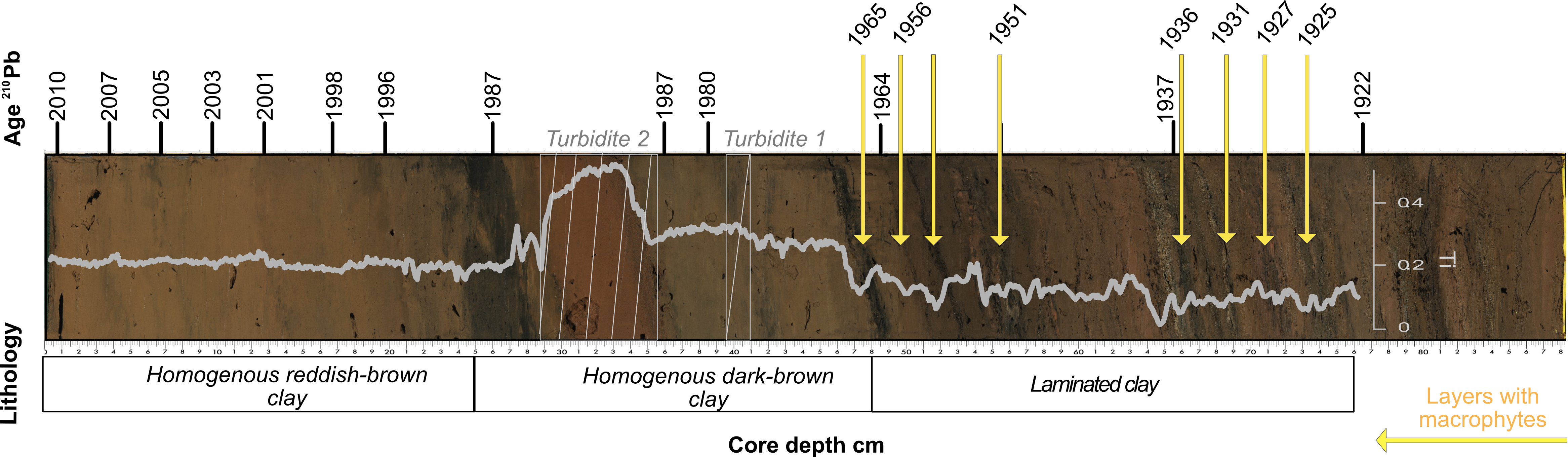

Supplement: S2 Fig — (TIFF) [file pone.0239209.s002.tiff]

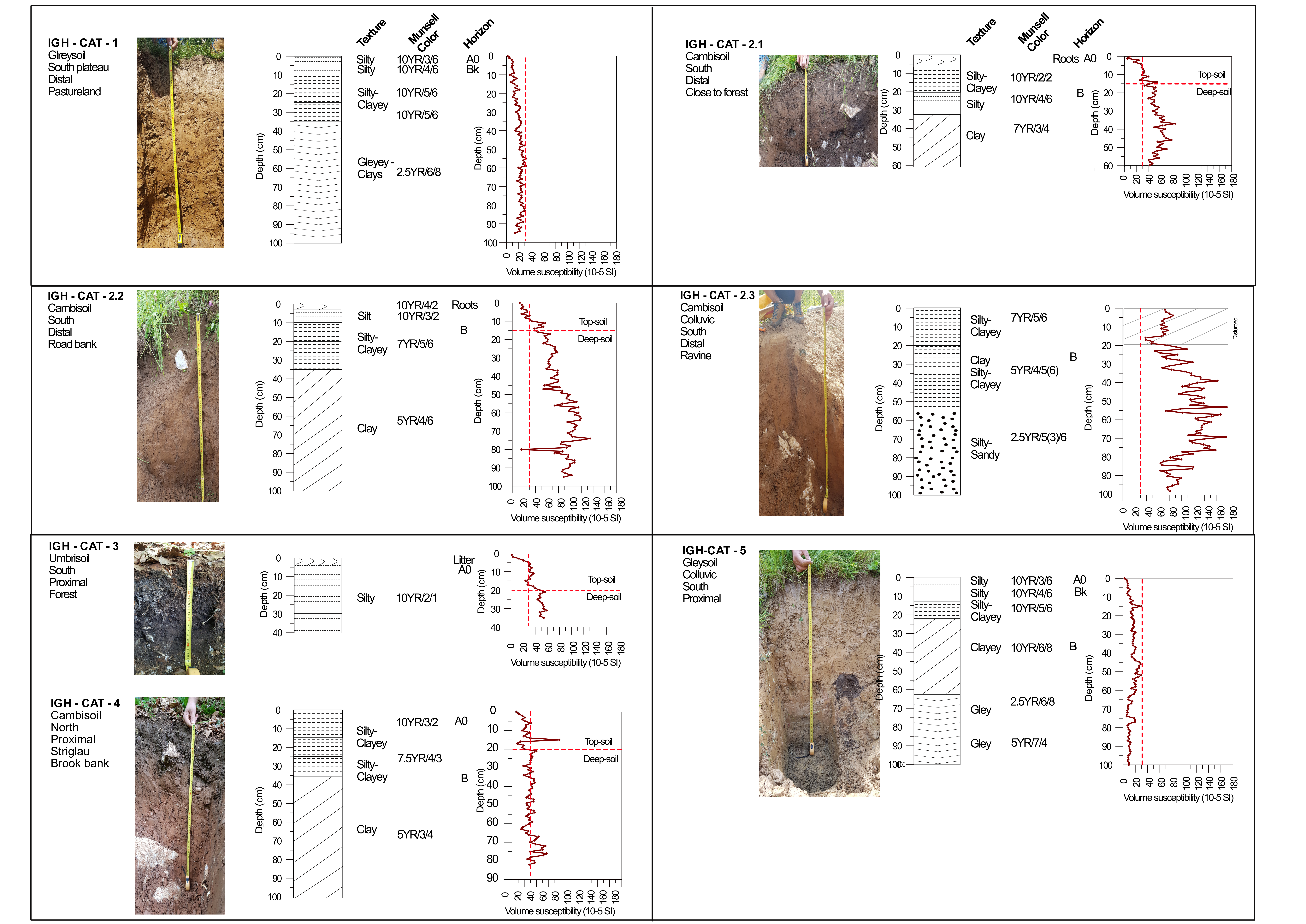

Supplement: S3 Fig — The location and picture of the soil profile is shown on the left side while the physical characteristics (composition, color), soil horizons and magnetic susceptibility is presented on the right side of each figure. Please note that soil type identification is based mainly on qualitative indices following their description following the national soil map. (TIFF) [file pone.0239209.s003.tiff]

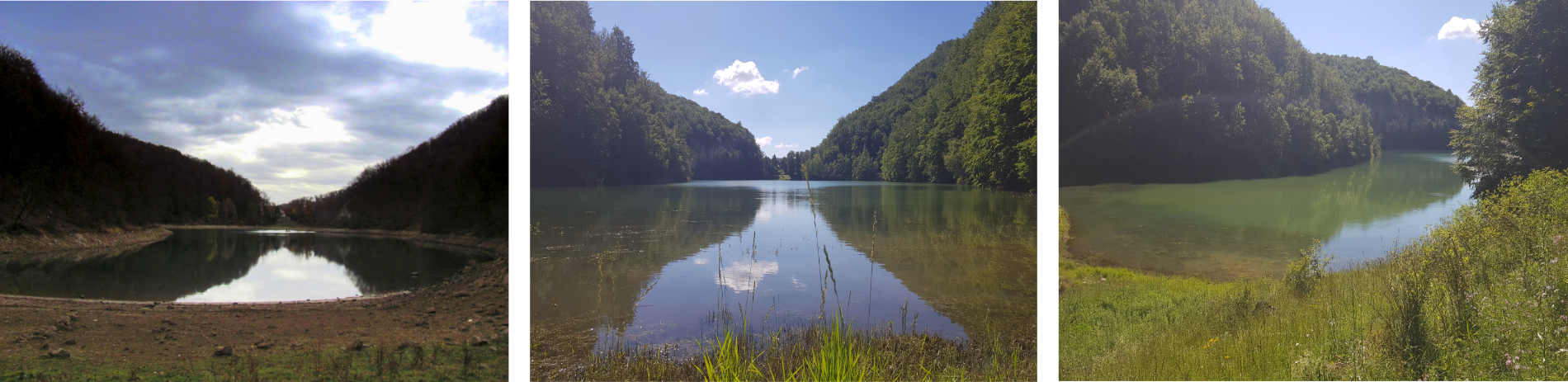

Supplement: S4 Fig — Photos taken from the north-western side of the lake in the Plesanului valley (photo courtesy of Daniel Veres and Aritina Haliuc). (TIFF) [file pone.0239209.s004.tiff]

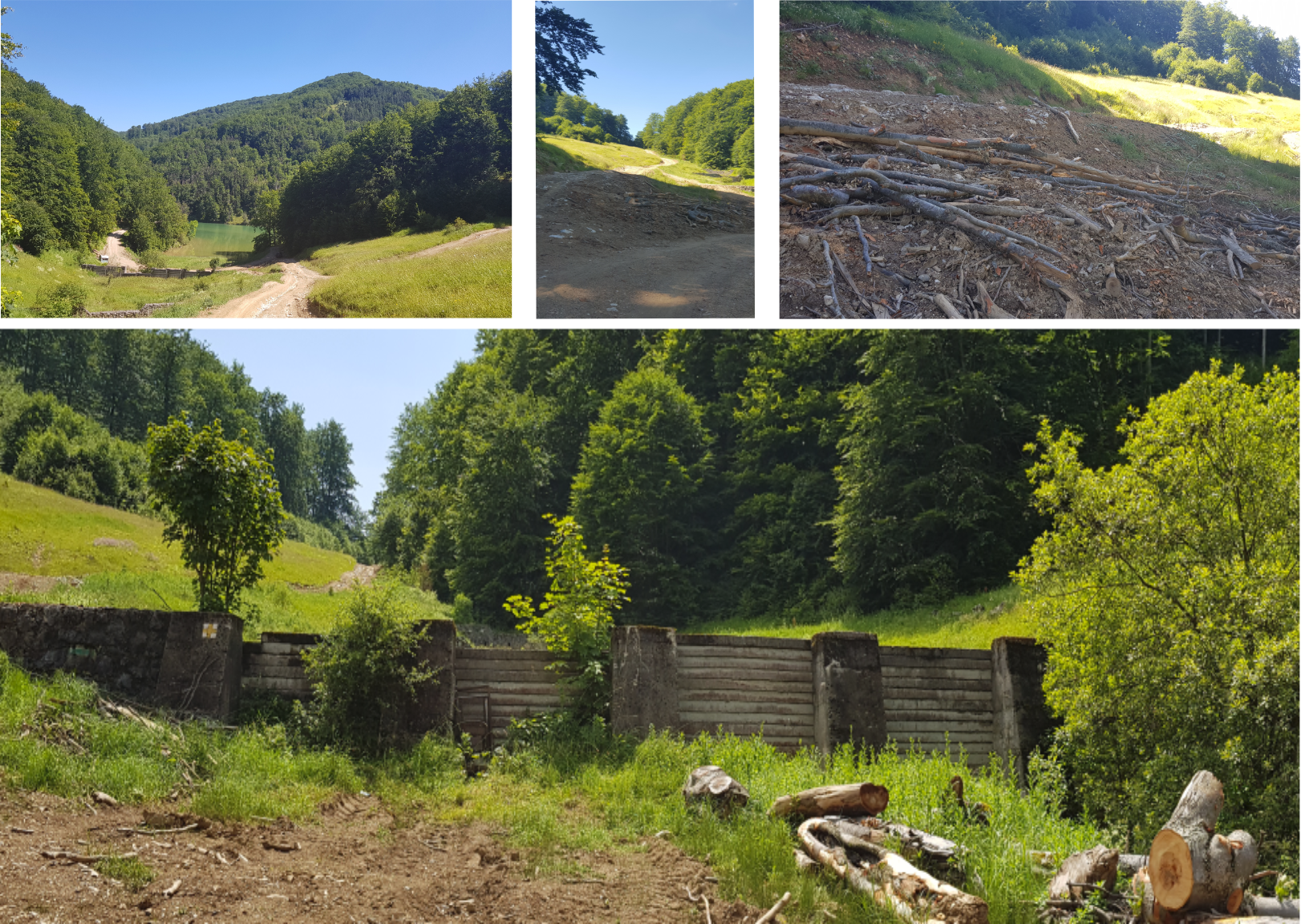

Supplement: S5 Fig — (TIFF) [file pone.0239209.s005.tiff]
