# Supplementary material for: Climate and land-use as the main drivers of recent environmental change in a mid-altitude mountain lake, Romanian Carpathians
Source: PLoS One. 2020 Oct 1;15(10):e0239209. doi: 10.1371/journal.pone.0239209 (PMC7529234; doi:10.1371/journal.pone.0239209)
Supplement: S1 Text — (DOCX) [file pone.0239209.s006.docx]

# **S1 Text Description of soil profiles from Ighiel catchment**

Please note that soil type is based on qualitative identification following the soil map of the area (coarse resolution). More detailed analysis is planned in the future. The main pedological types identified over the catchment include gleysols found mainly under the southern pasturelands, cambisols along the northern Striglau channel banks, while umbrisols outcrop in the proximity of the lake, in the forested area (S3 Fig; please see Fig 1, main text). The gleysol and colluvic cambisol profiles, IGH-CAT-1 and IGH-CAT-5 from the southern pastureland area show very low κ values, below 20-30 10^-5^ SI over the entire profile (please see main text, Table 1). The cambisol profiles IGH-CAT-2.1 to 2.3 collected along the southern ravine bank, show low κ values (<20-30 10^-5^ SI) for the 0-15 cm topsoil and slightly higher κ values (>30-40 10^-5^ SI) for the deep horizon (below 15-20 cm depth). A similar pattern is observed in the umbrisol IGH-CAT-3 profile, collected from the proximal forested area, with low κ values for the topsoil and high κ values for the deep soil part. The cambisol profile IGH-CAT-4, collected from the central-northern part of the catchment, the Striglau brook bank in the proximity of the lake, exhibit a similar trend, with κ values <20-30 10^-5^ SI for topsoil (above 20 cm depth) and slightly higher κ values 30-40 10^-5^ SI for deep soil (S3 Fig; please see main text, Table 1).

The red clay sample collected from the ravine (IGH-CAT-2) and the coarse, slope-wash material collected from the Striglau channel mouth, in the lake proximity (close to IGH-CAT-4) depict the highest κ values, 50-60 10^-5^ SI, while the fine, slope-wash pebbly sediment collected from the same point returned κ values close to 30 10^-5^ SI. The clay (deep weathering horizon) sample collected from the forest road, close to IGH-CAT-1, the sandstone sample and the fine and coarse slope-washed material collected close IGH-CAT-5 as well as the clay-sandy pellets collected from the Striglau channel mouth, in the lake proximity (close to IGH-CAT-4) all show lower κ values, <15 10^-5^ SI. The limestone sample returned negative (diamagnetic) κ values. Overall, the κ behaviour of the soil profiles shows that low κ values characterize erosion of the top-soil A horizon, while high κ values reflect deep soil erosion as seen in today in over deepen catchment channels and ravines draining towards the lake. The relatively low κ values for the IGH-CAT-1 and IGH-CAT-5 soil profiles likely reflect gleization, a chemical alteration of magnetic minerals in a moist clayey environment [1]. For ease of understanding, we categorise the κ data into two groups with high κ values showing deep soil, distal, channel erosion vs low κ values showing topsoil, proximal provenance (S3 Fig; please see main text, Table 1). These categories are fully concordant with our field observations on current dynamics for the riverine network draining into the lake.

**Reference**

1. Maher BA. Magnetic properties of modern soils and quaternary loessic paleosols: Paleoclimatic implications. Palaeogeography, Palaeoclimatology, Palaeoecology. 1998;137:25-54. https://doi.org/10.1016/S0031-0182(97)00103-X
