# Supplementary material for: Climate and land-use as the main drivers of recent environmental change in a mid-altitude mountain lake, Romanian Carpathians
Source: PLoS One. 2020 Oct 1;15(10):e0239209. doi: 10.1371/journal.pone.0239209 (PMC7529234; doi:10.1371/journal.pone.0239209)
Supplement: S1 Table — (DOCX) [file pone.0239209.s007.docx]

**S1 Table** The CRS model to calculate the age and the sedimentation rate for core SC-4

| **Depth** | **Concentration ^210^Pb (Bq/kg)** | **Conc. ^226^Ra (Bq/kg)** | **Uns. ^210^Pb (Bq/kg)** | **Cum. ^210^Pb** | **Age from now** | **Sed rate(g/cm2*y)** | **^137^Cs conc** |
| --- | --- | --- | --- | --- | --- | --- | --- |
|  |  |  | 0 | 0 | 2012 | 0 |  |
| 0.25 | 809.00 | 52.00 | 757.00 | 2334.42 | 2010.83 | 0.26 |  |
| 0.75 | 698.43 | 58.00 | 640.43 | 4349.88 | 2009.78 | 0.30 |  |
| 1.25 | 598.42 | 64.00 | 534.42 | 6094.71 | 2008.85 | 0.34 |  |
| 1.75 | 508.46 | 59.00 | 449.46 | 7035.60 | 2008.34 | 0.40 |  |
| 2.25 | 428.00 | 63.00 | 365.00 | 7770.56 | 2007.93 | 0.49 | 261 |
| 2.75 | 270.00 | 60.00 | 210.00 | 8537.69 | 2007.50 | 0.84 |  |
| 3.25 | 279.88 | 52.00 | 227.88 | 9057.58 | 2007.20 | 0.77 |  |
| 3.75 | 290.00 | 54.00 | 236.00 | 9751.84 | 2006.80 | 0.73 |  |
| 4.25 | 345.20 | 57.00 | 288.20 | 10223.03 | 2006.53 | 0.59 | 262 |
| 4.75 | 407.00 | 65.00 | 342.00 | 10851.93 | 2006.16 | 0.50 |  |
| 5.25 | 341.82 | 60.00 | 281.82 | 11489.36 | 2005.78 | 0.59 |  |
| 5.75 | 284.00 | 50.00 | 234.00 | 12191.98 | 2005.36 | 0.71 | 262 |
| 6.25 | 260.00 | 54.00 | 206.00 | 12674.01 | 2005.06 | 0.79 |  |
| 6.75 | 291.29 | 50.00 | 241.29 | 13182.27 | 2004.75 | 0.67 |  |
| 7.25 | 325.00 | 55.00 | 270.00 | 13845.15 | 2004.34 | 0.59 | 24 |
| 7.75 | 267.37 | 58.00 | 209.37 | 14380.86 | 2004.00 | 0.76 |  |
| 8.25 | 217.00 | 60.00 | 157.00 | 14843.45 | 2003.71 | 1.00 |  |
| 8.75 | 215.00 | 60.00 | 155.00 | 15206.99 | 2003.48 | 1.01 |  |
| 9.25 | 259.61 | 59.00 | 200.61 | 15548.71 | 2003.26 | 0.77 |  |
| 9.75 | 310.00 | 58.00 | 252.00 | 16042.23 | 2002.94 | 0.61 | 300 |
| 10.25 | 262.43 | 54.00 | 208.43 | 16521.47 | 2002.62 | 0.73 |  |
| 10.75 | 220.00 | 55.00 | 165.00 | 16864.47 | 2002.40 | 0.91 |  |
| 11.25 | 231.00 | 54.00 | 177.00 | 17264.76 | 2002.13 | 0.84 |  |
| 11.75 | 283.76 | 64.00 | 219.76 | 17675.10 | 2001.85 | 0.67 |  |
| 12.25 | 344.00 | 54.00 | 290.00 | 18318.09 | 2001.42 | 0.50 | 484 |
| 12.75 | 294.50 | 52.00 | 242.50 | 18950.00 | 2000.98 | 0.59 |  |
| 13.25 | 250.00 | 62.00 | 188.00 | 19444.88 | 2000.64 | 0.76 |  |
| 13.75 | 252.49 | 61.00 | 191.49 | 19860.45 | 2000.34 | 0.74 |  |
| 14.25 | 255.00 | 52.00 | 203.00 | 20269.18 | 2000.05 | 0.69 |  |
| 14.75 | 359.00 | 59.00 | 300.00 | 20926.99 | 1999.58 | 0.46 | 435 |
| 15.25 | 295.52 | 63.00 | 232.52 | 21374.56 | 1999.25 | 0.59 |  |
| 15.75 | 240.00 | 52.00 | 188.00 | 21844.46 | 1998.91 | 0.72 |  |
| 16.25 | 230.00 | 62.00 | 168.00 | 22328.24 | 1998.55 | 0.80 |  |
| 16.75 | 272.53 | 61.00 | 211.53 | 22656.13 | 1998.30 | 0.63 |  |
| 17.25 | 320.00 | 57.00 | 263.00 | 23263.30 | 1997.84 | 0.50 | 458 |
| 17.75 | 255.91 | 53.00 | 202.91 | 23634.61 | 1997.56 | 0.64 |  |
| 18.25 | 201.00 | 54.00 | 147.00 | 24010.57 | 1997.26 | 0.87 |  |
| 18.75 | 196.00 | 56.00 | 140.00 | 24341.66 | 1997.01 | 0.91 |  |
| 19.25 | 324.62 | 58.00 | 266.62 | 24886.12 | 1996.58 | 0.47 |  |
| 19.75 | 500.00 | 54.00 | 446.00 | 25680.13 | 1995.94 | 0.28 | 580 |
| 25.5 | 450.00 | 65.00 | 385.00 | 35246.90 | 1987.05 | 0.24 | 978 |
| 30.5 | 199.00 | 59.00 | 140.00 | 40937.74 | 1987.00 | 0.54 | 41 |
| 36.5 | 228.00 | 52.00 | 176.00 | 45751.96 | 1987.00 | 0.34 | 13 |
| 38.5 | 314.00 | 51.00 | 263.00 | 48169.55 | 1980.00 | 0.20 | 14 |
| 40.5 | 275.00 | 57.00 | 218.00 | 50806.45 | 1973.00 | 0.21 | 30 |
| 45.5 | 197.00 | 56.00 | 141.00 | 54601.97 | 1969.00 | 0.24 |  |
| 48.5 | 119.00 | 62.00 | 57.00 | 55608.66 | 1964.00 | 0.53 | 9 |
| 50.5 | 329.00 | 65.00 | 264.00 | 56538.99 | 1954.00 | 0.10 |  |
| 55.5 | 159.00 | 65.00 | 94.00 | 58950.69 | 1951.00 | 0.21 | 4 |
| 61.5 | 133.00 | 58.00 | 75.00 | 60461.72 | 1948.00 | 0.20 | 2 |
| 65.5 | 110.00 | 61.00 | 49.00 | 61251.06 | 1937.00 | 0.25 | 3 |
| 76.5 | 180.00 | 51.00 | 129.00 | 64230.54 | 1922.00 | 0.02 |  |
| 86.5 | 50.00 | 48.00 | 2.00 | 65247.57 |  |  |  |
