# Supplementary material for: Climate and land-use as the main drivers of recent environmental change in a mid-altitude mountain lake, Romanian Carpathians
Source: PLoS One. 2020 Oct 1;15(10):e0239209. doi: 10.1371/journal.pone.0239209 (PMC7529234; doi:10.1371/journal.pone.0239209)
Supplement: S2 Table — The taxonomical position of the diatoms was confirmed with the Algaebase [1]. The abbreviated name of the main taxa used in Fig 7 (main text) after [2]. (DOCX) [file pone.0239209.s008.docx]

**S2 Table** The most abundant diatoms in IGH-SC4 core. The taxonomical position of the diatoms was checked in Algaebase [1]. The abbreviated name of the main taxa used in Fig 7 (main text) after [2]

| **Diatoms Name and abbreviation** | **Reference** |
| --- | --- |
| Achnanthidium minutissimum (ADMI) | (Kützing) Czarnecki 1994 |
| Amphora copulata | (Kützing) Schoeman & R.E.M.Archibald 1986 |
| Asterionella formosa (AFOR) | Hassall 1850 |
| Brachysira neoexilis (BNEO) | Lange-Bertalot 1994 |
| Cymbella compacta | Østrup 1910 |
| Surirella librile (Syn: Cymatopleura solea) | (Ehrenberg) Ehrenberg 1845 |
| Denticula tenuis (DTEN) | Kützing 1844 |
| Diploneis oculata (DOCU) | (Brébisson) Cleve 1894 |
| Encyonopsis cesatii | (Rabenhorst) Krammer 1997 |
| Encyonopsis minuta (ECPM) | Krammer & E.Reichardt 1997 |
| Encyonopsis subminuta | Krammer & E.Reichardt 1997 |
| Eunotia arcubus (EARB) | Nörpel & Lange-Bertalot 1993 |
| Fragilaria capucina (FCAP) | Desmazières 1830 |
| Fragilaria mesolepta | Rabenhorst 1861 |
| Gomphonema spp |  |
| Gyrosigma acuminatum (GYAC) | (Kützing) Rabenhorst 1853 |
| Navicula cryptocephala | Kützing 1844 |
| Navicula cryptotenella (NCTE) | Lange-Bertalot 1985 |
| Navicula radiosa (NRAD) | Kützing 1844 |
| Nitzschia archibaldii (NIAR) | Lange-Bertalot 1980 |
| Nitzschia fonticola | (Grunow) Grunow 1881 |
| Nitzschia palea | (Kützing) W.Smith 1856 |
| Nitzschia recta (NREC) | Hantzsch ex Rabenhorst 1862 |
| Pantocsekiella costei (CCOS) | (J.C.Druart & F.Straub) K.T.Kiss & E.Ács 2016 |
| Pantocsekiella delicatula | (Hustedt) K.T.Kiss & E.Ács 2016 |
| Pantocsekiella ocellata (COCE) | (Pantocsek) K.T.Kiss & Ács 2016 |
| Planothidium frequentissimum | (Lange-Bertalot) Lange-Bertalot 1999 |
| Pseudostaurosira parasitica var. subconstricta | (Grunow) E.Morales 2003 |
| Sellaphora pupula | (Kützing) Mereschkovsky 1902 |
| Staurosirella pinnata (SPIN) | (Ehrenberg) D.M.Williams & Round 1988 |

**References**

1. Guiry MD, Guiry GM 2019. AlgaeBase [Internet]. World-wide electronic publication, National University of Ireland, Galway. 2019 [cited 2020 February 12]. Available from: [https://www.algaebase.org](https://www.algaebase.org/).
2. Rimet F, Bouchez A. Life-forms, cell-sizes and ecological guilds of diatoms in European rivers. Knowledge and Management of Aquatic Ecosystems. 2012;406(2012):01. https://doi.org/10.1051/kmae/2012018
